# Optimal timing to assess exercise‐induced oxidative stress: A systematic review and meta‐analysis

**DOI:** 10.1113/EP092963

**Published:** 2025-11-29

**Authors:** Chrysovalantis Stachteas, Nikolaos Georgogiannis, George G. Nastos, Panagiotis N. Chatzinikolaou, Petros C. Dinas, Anastasios A. Theodorou, Vassilis Paschalis, Ioannis S. Vrabas, Antonios Kyparos, Athanasios Z. Jamurtas, Ioannis G. Fatouros, Michalis G. Nikolaidis, Nikos V. Margaritelis

**Affiliations:** ^1^ Department of Physical Education and Sports Science at Serres Aristotle University of Thessaloniki Serres Greece; ^2^ School of Physical Education, Sport Science and Dietetics University of Thessaly Trikala Greece; ^3^ Department of Life Sciences, School of Sciences European University Cyprus Nicosia Cyprus; ^4^ School of Physical Education and Sport Science National and Kapodistrian University of Athens Athens Greece

**Keywords:** biomarkers, exercise, humans, oxidative stress, redox

## Abstract

There is a lack of consensus on optimal timing to assess redox biomarkers post‐exercise, limiting methodological standardisation and linking oxidative stress to physiology. We determined optimal post‐exercise oxidative stress assessment times using three redox biomarkers: glutathione, F_2_‐isoprostanes and protein carbonyls. Standardised mean differences were calculated using random‐effects models, with 95% confidence and prediction intervals. Risk of bias was assessed via RoB2 and ROBINS‐I tools. Egger's test and funnel plots evaluated publication bias. Certainty of evidence was rated using GRADE. PROSPERO preregistration: CRD42024508049. A total of 103 studies (*n* = 1418) were included. Glutathione levels decreased immediately (*g *= −0.70; 95% CI: −0.96, −0.44; *P *< 0.001), at 30 min to 2 h (*g *= −0.81; 95% CI: −1.19, −0.43; *P *< 0.001), and 48 h post‐exercise (*g *= −0.98; 95% CI: −1.50, −0.46; *P *< 0.01). F_2_‐isoprostanes increased immediately post‐exercise (*g *= 1.01; 95% CI: 0.70, 1.33; *P *< 0.001) and at 30 min to 2 h (*g *= 0.46; 95% CI: 0.23, 0.69; *P *< 0.001). Protein carbonyls increased at all time points, especially at 48 h post‐exercise (*g *= 1.17; 95% CI: 0.73, 1.60; *P *< 0.001), peaking at 72 h (*g *= 1.33; 95% CI: 0.52, 2.14; *P* = 0.0048). Subgroup analyses revealed that non‐muscle‐damaging exercise elicits responses immediately after exercise or within the first 2 h, while muscle‐damaging exercise induces peaks at 48 and 72 h post‐exercise. Egger's test indicated publication bias for F_2_‐isoprostanes (*P* = 0.017) and protein carbonyls (*P* = 0.031) post‐exercise. Risk of bias was moderate in randomised controlled trials and serious in non‐randomised studies. Certainty of evidence ranged from moderate to high. In conclusion, non‐muscle‐damaging exercise elicits early responses within hours, while muscle‐damaging protocols produce delayed peaks at 48–72 h. These findings support methodological consistency and are useful for optimising study design, sample size estimation and providing links between redox biology and physiological outcomes.

## Abstract (highlights)

- **What is the topic of this review?**
  The optimal timing to assess exercise‐induced oxidative stress in humans, via a systematic review and meta‐analysis of three commonly used redox biomarkers: glutathione (antioxidant), F_2_‐isoprostanes (lipid peroxidation product) and protein carbonyls (protein oxidation product).
- **What advances does it highlight?**
  It delineates biomarker‐ and protocol‐specific kinetics: non‐muscle‐damaging exercise peaks immediately/within 2 h, while muscle‐damaging protocols peak at 48–72 h. It offers specimen guidance, quantifies effect sizes, grades evidence and provides practical standards for study design.

## 1 INTRODUCTION

Redox biology has become increasingly recognised as a fundamental aspect of human biology, regulating, at least in part, both physiological and pathological conditions (Forman & Zhang, 2021; Jones & Sies, 2015; Sies et al., 2022). In the context of exercise science, research over the past 15 years has highlighted the role of redox processes, such as redox signalling and oxidative stress, in mediating key exercise responses (e.g., muscle fatigue) and adaptations (e.g., mitochondrial biogenesis) (Henriquez‐Olguin et al., 2023; Margaritelis et al., 2020b). To better understand and provide reliable explanations on the role of reactive species (e.g., hydrogen peroxide) and antioxidants (e.g., glutathione) in exercise physiology, the quantification of the diverse redox components has emerged as an essential requirement (Cobley, 2023; Forman et al., 2016; Jackson et al., 2020; Nikolaidis et al., 2020).

A prevailing challenge in modern research is the lack of methodological harmony across studies, which hampers the generation of comparable quantitative data. In redox biology, several seminal papers have underlined this limitation, predominantly from an analytical chemistry standpoint (Egea et al., 2017; Murphy et al., 2022; Winterbourn, 2014). Beyond these key analytical considerations, other methodological choices merit re‐evaluation. Following a long period characterised by ‘immature’ experimental approaches, such as the use of random antioxidant cocktails or the consumption of antioxidant‐rich foods to mitigate oxidative stress (Nikolaidis & Margaritelis, 2023), the field has now prioritised a shift toward rational methodological designs and justified experimental choices (Ghezzi & Mooradian, 2020; Lichtenberg et al., 2023; Margaritelis et al., 2022).

Five fundamental questions that every researcher has to address when designing a study are ‘who’, ‘what’, ‘how’, ‘where’ and ‘when’. The first four questions have been partially addressed in the redox biology literature, although they are still occasionally under debate. For instance, some studies suggest recruiting ‘rancid’ or antioxidant‐deficient individuals (‘who’; Halliwell, 2024; Margaritelis et al., 2020a), identifying and quantifying specific reactive species or systemic biomarkers associated with the redox pathway of interest (‘what’; Murphy et al., 2022; Sies et al., 2024), ensuring sample handling along with reliable and valid techniques (‘how’; Cobley et al., 2024; Poole et al., 2020) and exploiting recent advances in compartmentalised and highly localised dynamic sensing of redox processes (‘where’; Kano et al., 2024; Kritsiligkou et al., 2023). However, the question of ‘when’ is often subject to the discretion of researchers, who may choose time points for redox assessments based primarily on the characteristics of their available techniques rather than on a robust scientific rationale.

Meta‐analysis, as a powerful data‐based research synthesis method, may offer a valuable option to address this issue (Borenstein et al., 2021). Traditionally, meta‐analysis is used to synthesise evidence (e.g., calculating a summary effect size across studies), identify biases (e.g., publication and risk of bias), and estimate heterogeneity among studies (e.g., variability of true effect sizes, expressed typically as τ^2^). However, meta‐analysis also serves another crucial metascientific purpose by informing the design of future studies and suggesting improved research practices.

Accordingly, the primary aims of this systematic review and meta‐analysis were to identify the optimal timing to assess oxidative stress following acute exercise in humans. Two secondary aims were to investigate the impact of exercise type and biological specimen on exercise‐induced changes in oxidative stress levels. We focused on acute responses because time‐resolved biomarker kinetics define optimal sampling windows, reduce unnecessary sampling burden, and, most importantly, provide entry points to mechanistic interpretation of redox processes in exercise physiology. We anticipate that the findings from this meta‐analysis will provide valuable insights to guide future studies in exercise redox biology and promote methodological standardisation across the field.

## 2 METHODS

### 2.1 Design overview and search strategy

The protocol and the detailed search strategy have been preregistered on the International Prospective Register of Systematic Reviews (PROSPERO) database; registration number: CRD42024508049. As described within the preregistration protocol, we originally intended to include meta‐regression analyses examining the relationship between exercise‐induced oxidative stress and baseline antioxidant levels. Yet, this was not feasible since fewer than 10 studies, a predefined criterion, were identified that had measured all three biomarkers of interest. PubMed, EMBASE and Scopus were searched to identify all relevant studies. The initial search covered publications through June 2024, and a subsequent search expanded the database to include studies published up to April 2025. Additionally, the reference lists from the eligible studies and reviews relevant to our research question were screened to enrich the database. The search was limited to human studies, with no restrictions on publication date or language. All authors collaborated to develop the search algorithm. The initial search process was executed by the first three authors and reviewed by the last author. Due to a high number of potential keywords, we developed a keyword algorithm for each one of the outcomes (glutathione, F_2_‐isoprostanes and protein carbonyls). This allowed for a comprehensive searching procedure. The exact search algorithms per outcome for each one of the selected databases are provided in Supporting information, Table S1. This systematic review and meta‐analysis are reported in accordance with the Preferred Reporting Items for Systematic Reviews and Meta‐Analyses (PRISMA) guidelines and the relevant checklist is provided (Supporting information, Table S2) (Page et al., 2021).

### 2.2 Eligibility criteria for study selection

The studies identified were all complete reports published in peer‐reviewed journals. They were subsequently screened according to the PICOS methodology (Higgins et al., 2024) and the eligibility criteria were the following.

#### 2.2.1 Population

Healthy adults aged 18–65 years who are recreationally active, defined as individuals engaging in regular physical activity (e.g., exercise, sport, or fitness‐related activity), but who are not classified as competitive or elite athletes. Studies focusing on clinical populations or individuals with chronic diseases, as well as studies involving sedentary, aging (> 65 years) or underage (<18 years) participants, were excluded due to differences in resting redox status compared to healthy adults, as well as differences in exercise‐induced and antioxidant supplementation‐induced redox changes (Donato et al., 2010; Richardson et al., 2007).

#### 2.2.2 Intervention

Any type of acute exercise (i.e., single bout, event, match) without any restriction in duration, volume, intensity or fatigue occurrence, although it has been demonstrated that these variables may affect biomarker kinetics (Alessio et al., 2000; Quindry et al., 2003). When more than one exercise protocol was applied in a single study (e.g., two different protocols on an isokinetic dynamometer; Rosvoglou et al., 2023), the protocol that induced greater oxidative stress alterations was selected. When acute exercise was combined with a nutritional/dietary intervention (e.g., antioxidant supplementation), the data from the control ‘exercise‐only’ group were used.

#### 2.2.3 Comparator

The baseline or pre‐exercise resting oxidative stress biomarker levels.

#### 2.2.4 Outcome

The pre‐ to post‐exercise change in the levels of F_2_‐isoprostanes (lipid peroxidation; Nikolaidis et al., 2011), protein carbonyls (protein oxidation; Weber et al., 2015) and glutathione in its reduced form (i.e., GSH) unless otherwise specified (antioxidant; Deponte, 2013). We focused on these biomarkers because they are widely used in the literature as proxies of oxidative stress and have a stronger potential to capture acute exercise‐induced alterations in redox homeostasis compared to other frequently used biomarkers, such as TBARS/MDA and TAC, which have been widely critiqued for limited interpretational value in vivo and for analytical issues (e.g., poor specificity, susceptibility to artefactual formation, and high assay variability) (Kadiiska et al., 2005; Margaritelis, Cobley, et al., 2016). When studies evaluated oxidative stress at multiple time points up to 120 h post‐exercise, biomarker values at all time points were initially recorded (i.e., the main aim of the meta‐analysis). Moreover, studies using blood plasma, erythrocytes, skeletal muscle and/or urine samples were included in the meta‐analysis, irrespective of the analytical technique used in the original studies, although it is known that some assays, like commercially available kits, are less accurate, sensitive and specific compared to more refined techniques (Giustarini et al., 2013; Kano et al., 2025; Nikolaidis et al., 2011; Weber et al., 2015). For the sake of simplicity, we use the term ‘plasma’ even for studies in which serum was actually analysed.

#### 2.2.5 Study design

Both RCTs and non‐RCTs with parallel group or cross‐over design and single‐arm studies.

Based on the above eligibility criteria, animal studies, all types of review articles, study protocols, editorials, conference proceedings and grey literature publications were excluded.

### 2.3 Data extraction

A custom extraction spreadsheet was created in Microsoft Excel including study information (i.e., first author, year of publication, DOI), the sample size, a brief description of the acute exercise protocol (e.g., type, intensity, duration), the biological specimen (i.e., plasma, erythrocytes, skeletal muscle and/or urine) and the assayed biomarker (i.e., F_2_‐isoprostanes, protein carbonyls and/or glutathione). The above spreadsheet can be found in Supporting information, Table S3.

Data were collected as mean and standard deviation (SD). When the standard error of the mean (SEM) was presented in the original papers, it was first converted to standard deviation via the equation SD = SEM × sqrt(*n*). When data were presented only in a figure format, Web‐Plot Digitizer (Web Plot Digitizer, V.5.2. Texas, USA; Rohatgi A., 2024) was used to obtain raw values, and in some cases, personal communication with the authors was implemented. Data were extracted by at least two authors for each biomarker and a third author served as referee in the case of disagreement.

### 2.4 Data synthesis

For the meta‐analysis, a random‐effects model was applied, since we assumed that the true effect size varies between studies, with the respective effects being normally distributed, and that the studies in our analysis represent a random sample of effect sizes that could have been observed (Borenstein et al., 2021). Due to the different units reported in the original studies for the three outcome variables, we calculated the standardised mean difference (i.e., Hedges’ *g*) for each time point in all studies as well as the 95% confidence and prediction intervals, whilst the Knapp–Hartung adjustment was also applied (Hartung & Knapp, 2001). A positive Hedges’ *g* value indicated an increase in oxidative stress levels after acute exercise for F_2_‐isoprostanes and protein carbonyls, whilst a negative Hedges’ *g* value indicated an increase in oxidative stress levels after acute exercise for glutathione. Meta‐analysis was conducted when at least five studies were available for a specific time point, to ensure a sufficient level of statistical power, reduce the risk of biased estimates, and allow for a more meaningful assessment of heterogeneity. Although meta‐analyses can technically be performed with as few as two studies, reasonable thresholds are commonly used to improve the robustness and interpretability of the pooled results. Subgroup analyses were performed for the type of exercise (i.e., muscle‐damaging vs. non‐muscle‐damaging) and, when possible, for the biological specimen (i.e., plasma, erythrocytes, skeletal muscle and urine). When only one or two studies were found for a specimen at a particular time point, sensitivity analyses were conducted. We labelled an acute exercise session as muscle‐damaging under three conditions: (i) if explicitly stated by the authors in the original study (e.g., eccentric contractions on an isokinetic dynamometer); (ii) when the exercise type is widely recognised to include a strong eccentric component (e.g., downhill running); or (iii) if the original study assessed a biochemical or physiological marker of muscle damage (e.g., creatine kinase). The level of significance was set at α = 0.05. For the assessment of heterogeneity, we calculated the *Q*‐statistic and its *P*‐value, which serves as an estimate of the excess variance (i.e., evidence of heterogeneity); the τ^2^, which serves as the between‐studies variance of the true effects; and the *I* ^2^, which is the percent ratio of true heterogeneity between studies to total variation in the observed effects. All analyses were performed in R (version 4.4.2; R Core Team, 2024) and RStudio (2024.12.1+563; Posit Team, 2025) using custom scripts. The metafor (version 4.8‐0) package was used to calculate the study effect sizes, whilst meta (version 8.0‐2) was used to run the random‐effects models and create the forest plots (Schwarzer et al., 2015; Viechtbauer, 2010).

### 2.5 Risk of bias, publication bias and certainty of evidence

The Risk of Bias 2 (RoB2) tool and the ROBINS‐I tool from the Cochrane Library were used to assess the risk of bias in eligible randomised controlled trials and non‐randomised intervention studies, respectively (McGuinness & Higgins, 2021). To assess publication bias, we generated funnel plots and conducted Egger's tests in SPSS v.28 (IBM Corp., 2021) when at least 10 studies were available for a given biomarker at a specific time point (Borenstein et al., 2021). The certainty of evidence was assessed using the Grading of Recommendations Assessment, Development, and Evaluation (GRADE) analysis (Schünemann et al., 2013).

## 3 RESULTS

### 3.1 Overview of the literature search and study selection process

The initial search identified 3008, 3150 and 4304 publications for glutathione, F_2_‐isoprostanes and protein carbonyls, respectively. The subsequent screening process was identical for all three biomarkers. After removing duplicates, study titles were screened, followed by abstract and full‐text screening based on our predefined eligibility criteria. Ultimately, 41, 40 and 52 publications were included for glutathione, F_2_‐isoprostanes and protein carbonyls, respectively. Reference lists of the included studies were also examined, but no additional eligible publications were identified. A detailed PRISMA flow diagram for each biomarker is provided in Supporting information, Figures S1–S3. We meta‐analysed data at four time points for glutathione and F_2_‐isoprostanes (i.e., immediately, 30 min–2 h, 24 h and 48 h post‐exercise) and at five time points for protein carbonyls (i.e., immediately, 30 min–2 h, 24 h, 48 h and 72 h post‐exercise). Based on our predefined criteria, we did not analyse data at 12 h, 96 h and 120 h post‐exercise, as fewer than five studies were available for each biomarker at those time points (Allgrove et al., 2011; Bailey et al., 2011; Barbaresi et al., 2021; Berzosa et al., 2011; Bloomer et al., 2009; Broome et al., 2021; Ceci et al., 2015; Chaouachi et al., 2022; Chung et al., 2023; Silva et al., 2014; De Marchi et al., 2012, 2017; Deli et al., 2018; Deminice et al., 2013; Díaz‐Castro, Guisado, Kajarabille, García, Guisado, De Teresa, et al., 2012a; Díaz‐Castro, Guisado, Kajarabille, García, Guisado, de Teresa, et al., 2012b; Djordjevic et al., 2012; Draganidis et al., 2017; Filip‐Stachnik et al., 2023; Flanagan et al., 2018; Gholami et al., 2021; Goldfarb, Bloomer, et al., 2005a; Goldfarb et al., 2011; Goldfarb, Patrick, et al., 2005b; Gray et al., 2014; Howatson et al., 2010; Hudson et al., 2008; Jamurtas et al., 2018, 2006; Kaikkonen et al., 1998; Kalafati et al., 2010; Kerasioti et al., 2012; Knab et al., 2013; Kouvelioti et al., 2019; Kritikos et al., 2021; Leeder et al., 2014; Margaritelis et al., 2014, 2018; Margaritelis, Theodorou, et al., 2016; Martínez‐Noguera et al., 2019; Mastaloudis et al., 2004; McAllister et al., 2020; McAnulty et al., 2013; McAnulty et al., 2007c; McAnulty et al., 2003, 2008, 2010; McAnulty, Hosick, et al., 2007a; McAnulty, McAnulty, Nieman, et al., 2005b; McAnulty, McAnulty, Pascoe, et al., 2005a; McAnulty, Owens, et al., 2007d; McGinnis et al., 2014; Medved et al., 2004; Merry et al., 2010; Michailidis et al., 2013; Morillas‐Ruiz et al., 2005; Morrison et al., 2015; Mullins et al., 2013; Nakhostin‐Roohi et al., 2011; Nieman et al., 2013, 2004, 2016; Nieman, Scherr, et al., 2014b; Nieman, Shanely, et al., 2014a; Nikolaidis et al., 2012, 2013; Palazzetti et al., 2004; Panza et al., 2016; Panza et al., 2008; Papapanagiotou et al., 2011; Pappas et al., 2021; Paschalis et al., 2007, 2016; Pinto et al., 2022; Poulios et al., 2018; Quindry et al., 2013; Quindry et al., 2008; Quinn et al., 2021; Rietjens et al., 2007; Rosvoglou et al., 2023; Sacheck et al., 2003; Serravite et al., 2014; Silva et al., 2010, 2008; Skarpanska‐Stejnborn et al., 2008; Steensberg et al., 2002; Sureda et al., 2013; Tomazoni et al., 2019; Trewin et al., 2015, 2013; Wadley et al., 2017; White et al., 2013; Zembron‐Lacny et al., 2010, 2022).

### 3.2 Study characteristics

The 103 studies included in the meta‐analysis employed a wide range of exercise interventions, spanning from controlled, lab‐based protocols using isokinetic dynamometers, treadmills or cycle ergometers, to real‐world events such as official triathlons or football matches, as well as field‐based, sport‐specific fitness tests like the Yo‐Yo Test and the Loughborough Intermittent Shuttle Test (Supporting information, Table S3). Along with this diversity in the exercise protocols, most studies enrolled relatively small sample sizes, typically between 10 and 20 participants. However, only a few studies reported formal sample size calculations or provided a rationale for their chosen sample size, raising concerns about the statistical power and robustness of their findings. Notably, studies with larger samples were generally drawn from real‐world events, often utilising simple pre–post designs. Finally, the under‐representation of female participants remains a significant weakness in the exercise science literature (James et al., 2023), preventing us from conducting sex‐based subgroup analyses.

### 3.3 Risk of bias assessment

**FIGURE 1:**
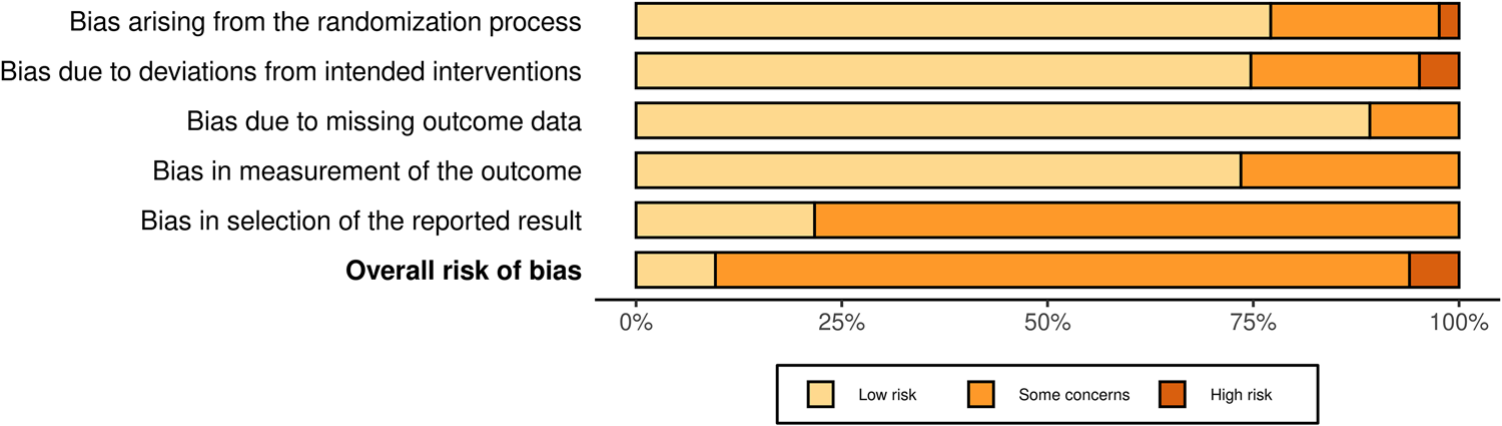
Summary of the overall risk of bias across included RCTs.

Regarding randomised controlled trials (RCTs), the majority of studies were rated as having an overall judgment of ‘some concerns’, primarily due to Domain 5 (‘bias in selection of the reported result’). This domain was commonly flagged because most studies lacked trial registration or a pre‐specified protocol. For the remaining domains (D1–D4), approximately three‐quarters of the studies were classified as ‘low risk’, whilst only five studies were rated as ‘high risk’ in Domain 1 and/or Domain 2. A summary of the overall risk of bias across studies is presented in Figure 1, while detailed individual assessments (i.e., traffic‐light plot) are provided in Supporting information, Figure S4.

**FIGURE 2:**
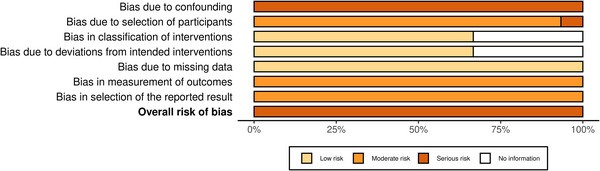
Summary of the overall risk of bias across non‐randomised or single‐arm intervention studies.

Regarding non‐randomised or single‐arm intervention studies, all studies in this group were rated as having an overall ‘serious risk’ of bias, driven almost exclusively by Domain 1 (‘bias due to confounding’). This rating was primarily due to the absence of randomisation and/or control groups, as well as the lack of statistical methods to adjust for baseline differences or time‐varying confounders. These limitations greatly reduce confidence in attributing observed effects solely to the intervention. Across the remaining six domains of the tool (D2–D7), almost all studies were rated as having a ‘moderate risk’ of bias in Domains 2, 6 and 7, each for various study‐specific reasons (Figure 2). Detailed risk of bias assessments for individual studies (i.e., traffic light plot) are available in Supporting information, Figure S5. One study (Quinn et al., 2021) could not be assessed using either the RoB 2 or ROBINS‐I tools, as the design did not align with the criteria required for randomised or non‐randomised intervention studies (i.e., reliability of a point‐of‐care device). Thus, this study was assessed with the COSMIN Risk of Bias checklist (Mokkink et al., 2018), and it showed low risk of bias across key domains for reliability studies, indicating a robust and well‐conducted evaluation.

### 3.4 Meta‐analysis results

#### 3.4.1 Glutathione

**FIGURE 3:**
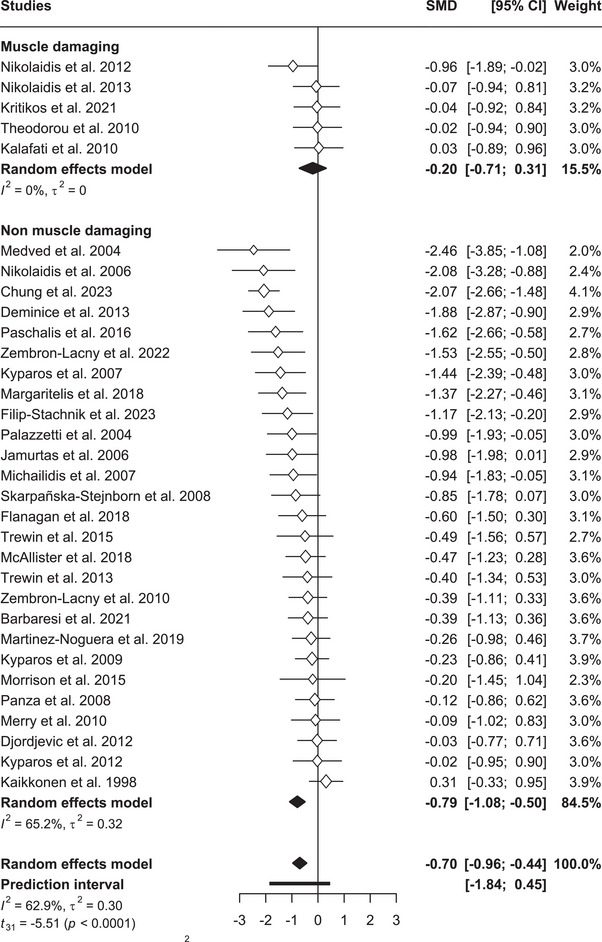
Forest plot of the effects of acute exercise on glutathione levels immediately post‐exercise.

A total of 41 studies (*n* = 572 participants) were included in the meta‐analysis examining the effect of acute exercise on glutathione levels. Immediately post‐exercise (*k* = 32, *n* = 378), the overall pooled effect size was *g* = −0.70 (95% CI: −0.96, −0.44; *t*_31_ = −5.51, *P* < 0.001), indicating a decrease in glutathione levels and an increase in oxidative stress. Regarding the level of heterogeneity across studies, we found *I* ^2^ = 62.9%, τ^2^ = 0.30, *Q*(31) = 83.62 and *P* < 0.001, suggesting that differences in effect sizes across studies may be partially attributed to study‐level characteristics. Subgroup analyses were conducted based on the type of exercise (muscle‐damaging vs. non‐muscle‐damaging), which indicated that the effect was larger in non‐muscle‐damaging studies (*k* = 27) (*g* = −0.79; 95% CI: −1.08, −0.50) compared to muscle‐damaging studies (*k* = 5), which showed a small and non‐significant effect (*g* = −0.20; 95% CI: −0.71, 0.31) (Figure 3).

**FIGURE 4:**
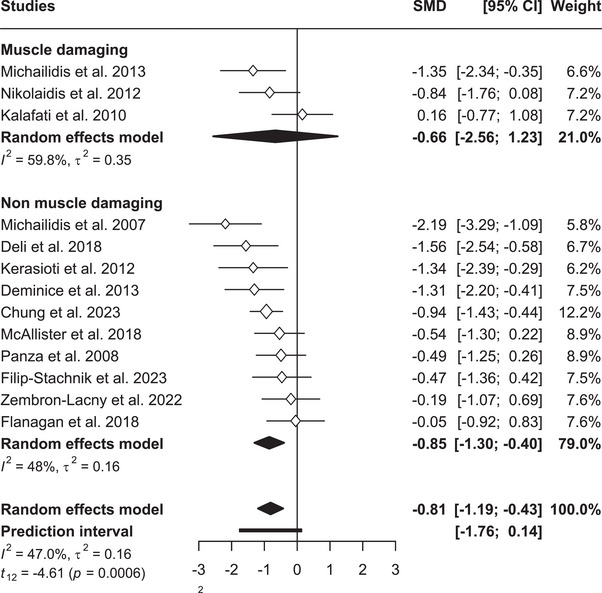
Forest plot of the effects of acute exercise on glutathione levels 30 min to 2 h post‐exercise.

Regarding 30 min to 2 h post‐exercise (*k* = 13, *n* = 165), the overall pooled effect size was *g* = −0.81 (95% CI: −1.19, −0.43; *t*_12_ = −4.61, *P* < 0.001), indicating a decrease in glutathione levels and an increase in oxidative stress. Regarding the level of heterogeneity across studies, we found *I* ^2^ = 47%, τ^2^ = 0.16, *Q*(12) = 22.65 and *P* = 0.031, suggesting that differences in effect sizes across studies may be partially attributed to study‐level characteristics. Subgroup analyses were conducted based on the type of exercise (muscle‐damaging vs. non‐muscle‐damaging), which indicated that the effect was larger in non‐muscle‐damaging studies (*k* = 10) (*g* = −0.85; 95% CI: −1.30, −0.40) compared to muscle‐damaging studies (*k* = 3), which showed a non‐significant effect (*g* = −0.66; 95% CI: −2.56, 1.23) (Figure 4).

**FIGURE 5:**
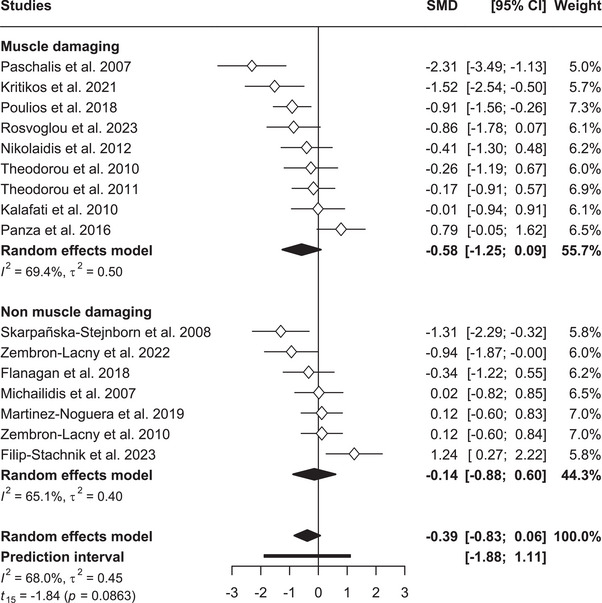
Forest plot of the effects of acute exercise on glutathione levels 24 h post‐exercise.

Regarding 24 h post‐exercise (*k* = 16, *n* = 185), the overall pooled effect size was *g* = −0.39 (95% CI: −0.83, 0.06; *t*_15_ = −1.84, *P* = 0.086), indicating a non‐significant effect of acute exercise on glutathione levels. Regarding the level of heterogeneity across studies, we found *I* ^2^ = 68%, τ^2^ = 0.45, *Q*(15) = 46.87 and *P* < 0.001, suggesting that differences in effect sizes across studies may be partially attributed to study‐level characteristics. Subgroup analyses were conducted based on the type of exercise (muscle‐damaging vs. non‐muscle‐damaging), which indicated that the effect was non‐significant in both non‐muscle‐damaging (*k* = 7) (*g* = −0.14; 95% CI: −0.88, 0.60) and muscle‐damaging studies (*k* = 9) (*g* = −0.58; 95% CI: −1.25, 0.09) (Figure 5).

**FIGURE 6:**
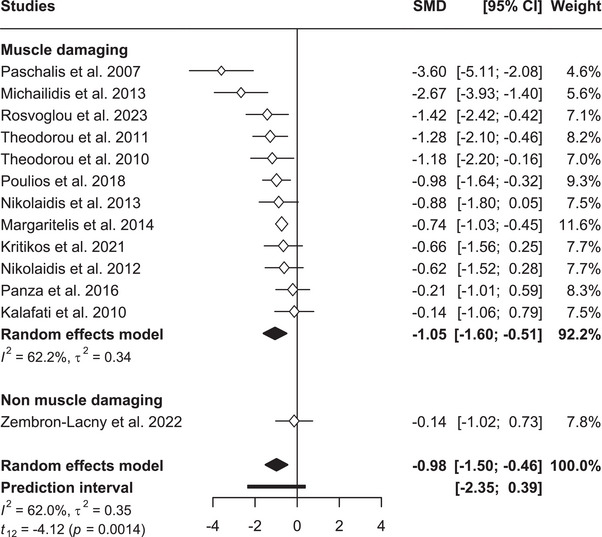
Forest plot of the effects of acute exercise on glutathione levels 48 h post‐exercise.

Regarding 48 h post‐exercise (*k* = 13, *n* = 232), the overall pooled effect size was *g* = −0.98 (95% CI: −1.50, −0.46; *t*_12_ = −4.12, *P* = 0.0014), indicating a decrease in glutathione levels and an increase in oxidative stress. Regarding the level of heterogeneity across studies, we found *I* ^2^ = 62%, τ^2^ = 0.35, *Q*(12) = 31.58 and *P* = 0.0016, suggesting that differences in effect sizes across studies may be partially attributed to study‐level characteristics. Subgroup analyses were conducted based on the type of exercise (muscle‐damaging vs. non‐muscle‐damaging), which indicated a large effect in muscle‐damaging studies (*k* = 12) (*g* = −1.05; 95% CI: −1.60, −0.51), whilst only one study was found with a non‐muscle‐damaging protocol (Figure 6).

All studies measuring glutathione levels in blood plasma, serum or urine were excluded from the meta‐analysis, as these matrices lack biological relevance for evaluating glutathione concentration and/or activity. Glutathione primarily functions intracellularly; therefore, only human studies that measured glutathione in erythrocytes or skeletal muscle biopsies were included. Subgroup analyses based on the biological specimen were planned, but a sufficient number of studies for comparison was available only at the immediate post‐exercise time point (i.e., a single skeletal muscle study was available for the other time points). At this time point, subgroup analysis revealed a significant effect in erythrocyte studies (*k* = 28; *g* = −0.70; 95% CI: −0.97, −0.43), in contrast to the non‐significant effect observed in skeletal muscle studies (*k* = 4; *g* = −0.74; 95% CI: −2.44, 0.95) (Supporting information, Figure S6).

#### 3.4.2 F_2_‐isoprostanes

**FIGURE 7:**
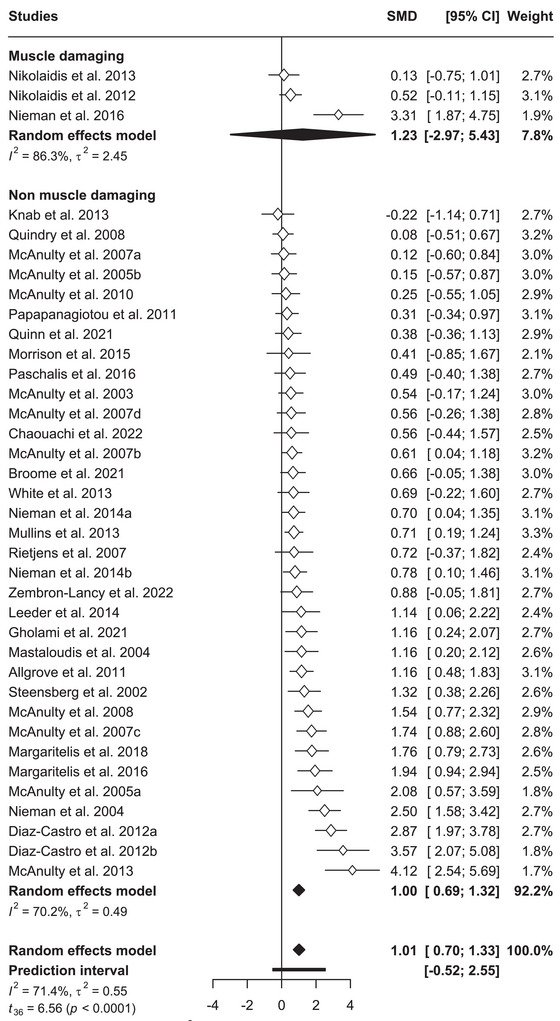
Forest plot of the effects of acute exercise on F_2_‐isoprostanes immediately post‐exercise.

A total of 40 studies (*n* = 635 participants) were included in the meta‐analysis examining the effect of acute exercise on F_2_‐isoprostanes. Immediately post‐exercise (*k* = 37, *n* = 511), the overall pooled effect size was *g* = 1.01 (95% CI: 0.70, 1.33; *t*_36_ = 6.56, *P* < 0.001), indicating an increase in F_2_‐isoprostanes and oxidative stress. Regarding the level of heterogeneity across studies, we found *I* ^2^ = 71.4%, τ^2^ = 0.55, *Q*(36) = 125.71 and *P* < 0.001, suggesting that differences in effect sizes across studies may be partially attributed to study‐level characteristics. Subgroup analyses were conducted based on the type of exercise (muscle‐damaging vs. non‐muscle‐damaging), which indicated a large effect in non‐muscle‐damaging studies (*k* = 34) (*g* = 1.00; 95% CI: 0.69, 1.32) compared to muscle‐damaging studies (*k* = 3), which showed a non‐significant effect (*g* = 1.23; 95% CI: −2.97, 5.43) (Figure 7).

**FIGURE 8:**
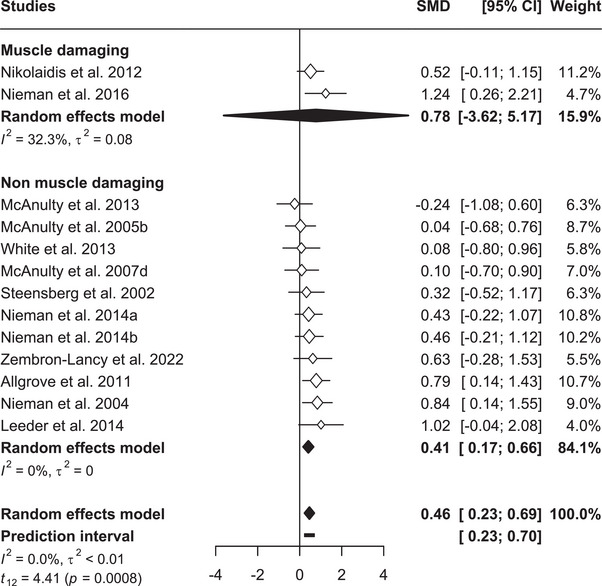
Forest plot of the effects of acute exercise on F_2_‐isoprostanes 30 min to 2 h post‐exercise.

Regarding 30 min to 2 h post‐exercise (*k* = 13, *n* = 181), the overall pooled effect size was *g* = 0.46 (95% CI: 0.23, 0.69; *t*_12_ = 4.41, *P* < 0.001), indicating an increase in F_2_‐isoprostanes and oxidative stress. There was no evidence of heterogeneity among the studies, since we found *I* ^2^ = 0%, τ^2^ < 0.01, *Q*(12) = 11.39 and *P* = 0.496, suggesting that the variability in effect sizes was compatible with random sampling error. Subgroup analyses were conducted based on the type of exercise (muscle‐damaging vs. non‐muscle‐damaging), which indicated that the effect was significant in non‐muscle‐damaging studies (*k* = 11) (*g* = 0.41; 95% CI: 0.17, 0.66) compared to muscle‐damaging studies (*k* = 2), which showed a non‐significant effect (*g* = 0.78; 95% CI: −3.62, 5.17) (Figure 8).

**FIGURE 9:**
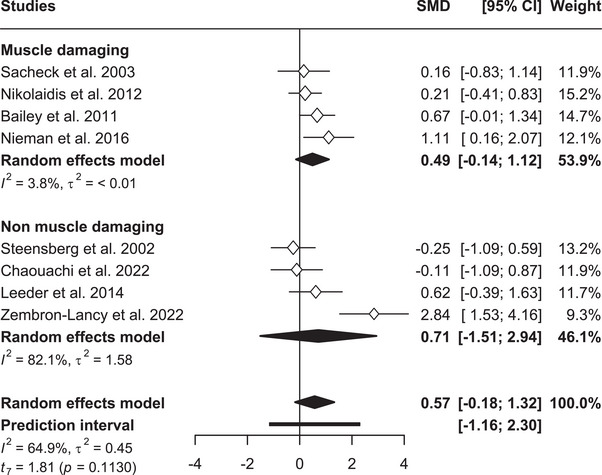
Forest plot of the effects of acute exercise on F_2_‐isoprostanes 24 h post‐exercise.

Regarding 24 h post‐exercise (*k* = 8, *n* = 93), the overall pooled effect size was *g* = 0.57 (95% CI: −0.18, 1.32; *t*_7_ = 1.81, *P* = 0.113), indicating a non‐significant effect of acute exercise on F_2_‐isoprostane levels. Regarding the level of heterogeneity across studies, we found *I* ^2^ = 64.9%, τ^2^ = 0.45, *Q*(7) = 19.94 and *P* < 0.01, suggesting that differences in effect sizes across studies may be partially attributed to study‐level characteristics. Subgroup analyses were conducted based on the type of exercise (muscle‐damaging vs. non‐muscle‐damaging), which indicated that the effect was non‐significant in both non‐muscle‐damaging (*k* = 4) (*g* = 0.71; 95% CI: −1.51, 2.94) and muscle‐damaging studies (*k* = 4) (*g* = 0.49; 95% CI: −0.14, 1.12) (Figure 9).

**FIGURE 10:**
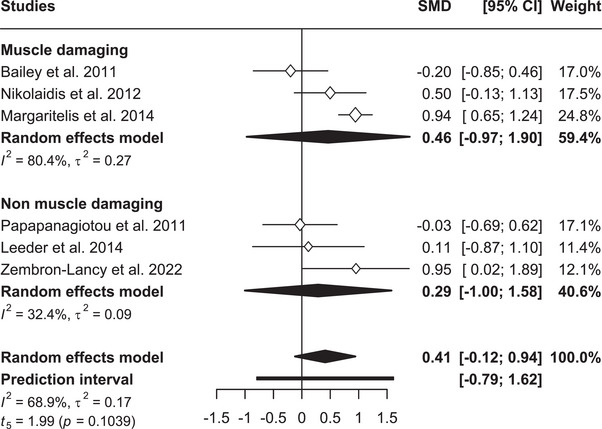
Forest plot of the effects of acute exercise on F_2_‐isoprostanes 48 h post‐exercise.

Regarding 48 h post‐exercise (*k* = 6, *n* = 172), the overall pooled effect size was *g* = 0.41 (95% CI: −0.12, 0.94; *t*_5_ = 1.99, *P* = 0.104), indicating a non‐significant effect of acute exercise on F_2_‐isoprostane levels. Regarding the level of heterogeneity across studies, we found *I* ^2^ = 68.9%, τ^2^ = 0.17, *Q*(5) = 16.06 and *P* < 0.01, suggesting that differences in effect sizes across studies may be partially attributed to study‐level characteristics. Subgroup analyses were conducted based on the type of exercise (muscle‐damaging vs. non‐muscle‐damaging), which indicated that the effect was non‐significant in both non‐muscle‐damaging (*k* = 3) (*g* = 0.29; 95% CI: −1.00, 1.58) and muscle‐damaging studies (*k* = 3) (*g* = 0.46; 95% CI: −0.97, 1.90) (Figure 10).

Subgroup analyses based on the biological specimen were planned, but a sufficient number of studies for this comparison was available only at the immediate post‐exercise time point. At this time point, subgroup analysis revealed a significant effect both in plasma (*k* = 280 (*g* = 0.91; 95% CI: 0.58, 1.23) and urine studies (*k* = 8) (*g* = 1.43; 95% CI: 0.42, 2.45) (Supporting information, Figure S7).

#### 3.4.3 Protein carbonyls

**FIGURE 11:**
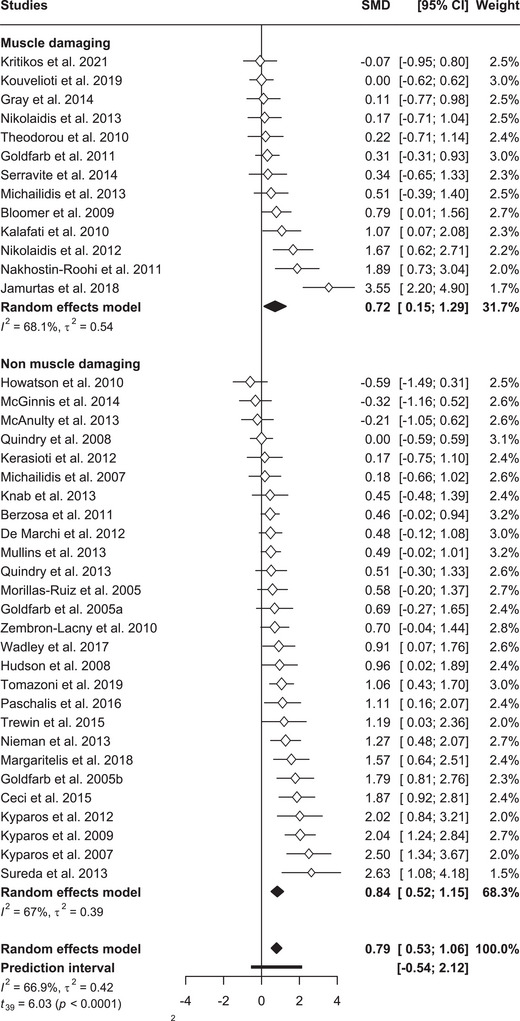
Forest plot of the effects of acute exercise on protein carbonyls immediately post‐exercise.

A total of 52 studies (*n* = 751 participants) were included in the meta‐analysis examining the effect of acute exercise on protein carbonyls. Immediately post‐exercise (*k* = 40, *n* = 528), the overall pooled effect size was *g* = 0.79 (95% CI: 0.53, 1.06; *t*_39_ = 6.03, *P* < 0.001), indicating an increase in protein carbonyls and oxidative stress. Regarding the level of heterogeneity across studies, we found *I* ^2^ = 66.9%, τ^2^ = 0.42, *Q(*39) = 117.99 and *P* < 0.001, suggesting that differences in effect sizes across studies may be partially attributed to study‐level characteristics. Subgroup analyses were conducted based on the type of exercise (muscle‐damaging vs. non‐muscle‐damaging), which indicated a large effect in both non‐muscle‐damaging (*k* = 27) (*g* = 0.84; 95% CI: 0.52, 1.15) and muscle‐damaging studies (*k* = 13) (*g* = 0.72; 95% CI: 0.15, 1.29) (Figure 11).

**FIGURE 12:**
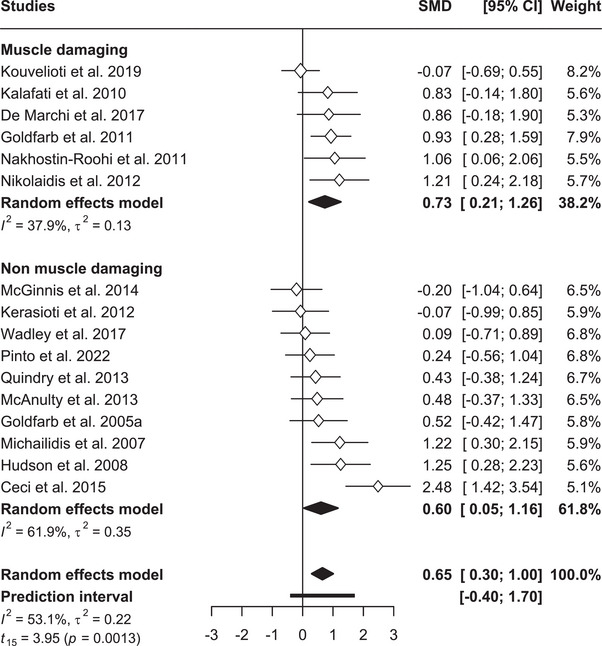
Forest plot of the effects of acute exercise on protein carbonyls 30 min to 2 h post‐exercise.

Regarding 30 min to 2 h post‐exercise (*k* = 16, *n* = 186), the overall pooled effect size was *g* = 0.65 (95% CI: 0.30, 1.01; *t*_15_ = 3.95, *P* < 0.01), indicating an increase in protein carbonyls and oxidative stress. Regarding the level of heterogeneity across studies, we found *I* ^2^ = 53.1%, τ^2^ = 0.22, *Q*(15) = 31.99, *P* < 0.01, suggesting that differences in effect sizes across studies may be partially attributed to study‐level characteristics. Subgroup analyses were conducted based on the type of exercise (muscle‐damaging vs. non‐muscle‐damaging), which indicated that the effect was larger in muscle‐damaging studies (*k* = 6) (*g* = 0.73; 95% CI: 0.21, 1.26) compared to non‐muscle‐damaging studies (*k* = 10) (*g* = 0.60; 95% CI: 0.05, 1.16) (Figure 12).

**FIGURE 13:**
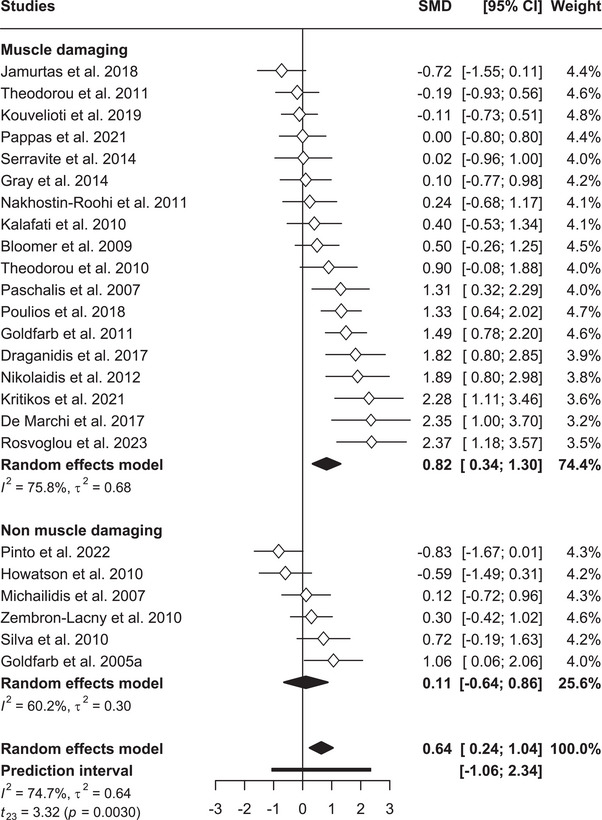
Forest plot of the effects of acute exercise on protein carbonyls 24 h post‐exercise.

Regarding 24 h post‐exercise (*k* = 24, *n* = 283), the overall pooled effect size was *g* = 0.64 (95% CI: 0.24, 1.04; *t*_23_ = 3.32, *P* < 0.01), indicating an increase in protein carbonyls and oxidative stress. Regarding the level of heterogeneity across studies, we found *I* ^2^ = 74.7%, τ^2^ = 0.64, *Q*(23) = 90.91 and *P* < 0.010, suggesting that differences in effect sizes across studies may be partially attributed to study‐level characteristics. Subgroup analyses were conducted based on the type of exercise (muscle‐damaging vs. non‐muscle‐damaging), which indicated that the effect was significant in muscle‐damaging studies (*k* = 18) (*g* = 0.82; 95% CI: 0.34, 1.30) compared to non‐muscle‐damaging studies (*k* = 6) which showed a non‐significant effect (*g* = 0.11; 95% CI: −0.64, 0.86) (Figure 13).

**FIGURE 14:**
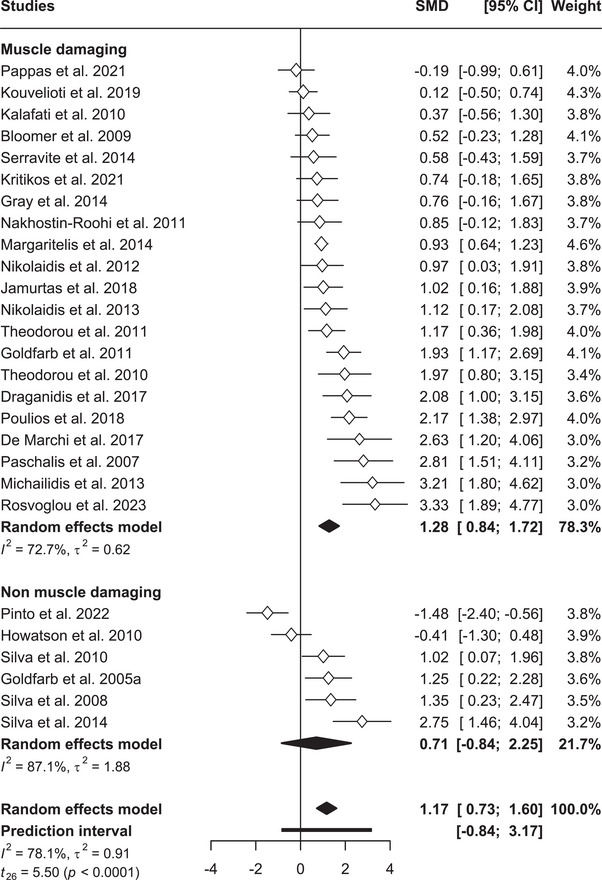
Forest plot of the effects of acute exercise on protein carbonyls 48 h post‐exercise.

Regarding 48 h post‐exercise (*k* = 27, *n* = 393), the overall pooled effect size was *g* = 1.17 (95% CI: 0.73, 1.60; *t*_26_ = 5.50, *P* < 0.001), indicating an increase in protein carbonyls and oxidative stress. Regarding the level of heterogeneity across studies, we found *I* ^2^ = 78.1%, τ^2^ = 0.91, *Q*(26) = 118.82 and *P* < 0.001, suggesting that differences in effect sizes across studies may be partially attributed to study‐level characteristics. Subgroup analyses were conducted based on the type of exercise (muscle‐damaging vs. non‐muscle‐damaging), which indicated that the effect was significant in muscle‐damaging studies (*k* = 21) (*g* = 1.28; 95% CI: 0.84, 1.72) compared to non‐muscle‐damaging studies (*k* = 6), which showed a non‐significant effect (*g* = 0.71; 95% CI: −0.84, 2.25) (Figure 14).

**FIGURE 15:**
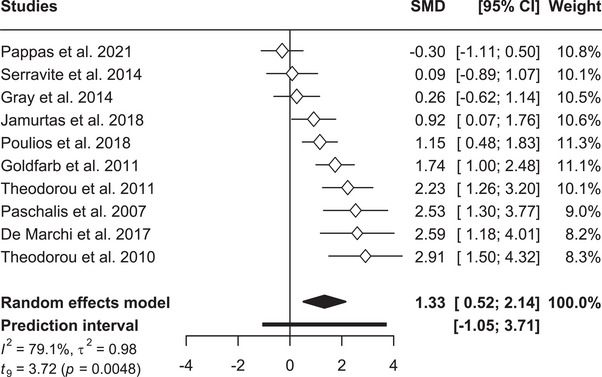
Forest plot of the effects of acute exercise on protein carbonyls 72 h post‐exercise.

Regarding 72 h post‐exercise (*k* = 10, *n* = 123), the overall pooled effect size was *g* = 1.33 (95% CI: 0.52, 2.14; *t*_9_ = 3.72, *P* = 0.0048), indicating an increase in protein carbonyls and oxidative stress. Regarding the level of heterogeneity across studies, we found *I* ^2^ = 79.1%, τ^2^ = 0.98, *Q*(9) = 43.15 and *P* < 0.001, suggesting that differences in effect sizes across studies may be partially attributed to study‐level characteristics. Subgroup analyses were not conducted since all included studies at this time point applied muscle‐damaging exercise protocols (Figure 15).

No subgroup analyses based on the biological specimen were conducted, since almost all studies used blood plasma for protein carbonyls measurement, except for two studies that used erythrocytes, two studies that used skeletal muscle and one study that used neutrophils.

### 3.5 Publication bias assessment

Publication bias was assessed using Egger's test and visual inspection of funnel plots (Supporting information, Table S4). For glutathione, Egger's tests revealed no significant asymmetry in any time point or subgroup with adequate data. Specifically, overall effects immediately post‐exercise (*k* = 32, *P* = 0.415), 30 min–2 h (*k* = 13, *P* = 0.656), 24 h (*k* = 16, *P* = 0.209) and 48 h post‐exercise (*k* = 13, *P* = 0.352) all showed non‐significant results. Subgroup analyses by exercise type similarly showed no signs of bias (*P *> 0.05 in all time points), except where Egger's test was not applicable due to limited studies (*k* < 10). Similarly, the funnel plots for glutathione appear symmetrical across all time points, supporting the Egger's test results that showed no evidence of bias. Data points are evenly distributed around the central effect estimate, and small studies do not disproportionately cluster on one side. It is possible that glutathione, due to its more dynamic metabolism (i.e., production, regeneration, cellular efflux, etc.), shows less dramatic and more consistent changes post‐exercise compared to oxidative stress biomarkers like protein carbonyls or F_2_‐isoprostanes, leading to a more balanced publication landscape.

In contrast, F_2_‐isoprostanes showed some signs of publication bias immediately post‐exercise (*k* = 37, *P* = 0.017), especially for non‐muscle‐damaging protocols (*k* = 34, *P* = 0.049). However, no bias was detected at later time points (30 min–2 h, 24 h or 48 h), though statistical tests were often non‐applicable due to the small number of studies. These results suggest a potential small‐study effect influencing early post‐exercise F_2_‐isoprostane findings. For protein carbonyls, evidence of publication bias was also detected at some time points. Egger's test was significant immediately post‐exercise overall (*k* = 40, *P* = 0.031), particularly in studies using muscle‐damaging exercise (*k* = 13, *P* = 0.013), and at 24 h (*k* = 24, *P* = 0.031). No significant bias was observed at 30 min–2 h, 48 h or 72 h post‐exercise. The funnel plots for both F_2_‐isoprostanes and protein carbonyls immediately post‐exercise showed a clear asymmetry, which backs up Egger's test results. Across all biomarkers, subgroup analyses at time points with fewer than 10 studies were considered underpowered, and Egger's test was not applied in those instances

### 3.6 Certainty of evidence assessment

The summary outcomes of the GRADE analysis can be found in Table 1, while the detailed GRADE process for each meta‐analysis can be found in Supporting information, Supplementary File 3.

**TABLE 1:** GRADE analysis.

Biomarker	*n* (*k*)	Certainty of evidence	Comment
Glutathione			
Immediately post	378 (32)	High ⨁⨁⨁⨁	We are very confident that the true effect lies close to that of the estimate of the effect
30 min–2 h post	165 (13)	High ⨁⨁⨁⨁	We are very confident that the true effect lies close to that of the estimate of the effect
48 h post	232 (13)	High ⨁⨁⨁⨁	We are very confident that the true effect lies close to that of the estimate of the effect
F_2_‐isoprostanes			
Immediately post	511 (37)	Moderate ⨁⨁⨁◯ Due to the risk of bias	We are moderately confident in the effect estimate: the true effect is likely to be close to the estimate of the effect, but there is a possibility that it is different
30 min–2 h post	181 (13)	Low ⨁⨁◯◯ Due to the risk of bias	Our confidence in the effect estimate is limited: the true effect may be substantially different from the estimate of the effect
Protein carbonyls			
Immediately post	528 (40)	Low ⨁⨁◯◯ Due to the risk of bias and publication bias	Our confidence in the effect estimate is limited: the true effect may be substantially different from the estimate of the effect
30 min–2 h post	186 (16)	Moderate ⨁⨁⨁◯ Due to the risk of bias	We are moderately confident in the effect estimate: the true effect is likely to be close to the estimate of the effect, but there is a possibility that it is different
24 h post	283 (24)	Low ⨁⨁◯◯ Due to the risk of bias and publication bias	Our confidence in the effect estimate is limited: the true effect may be substantially different from the estimate of the effect
48 h post	393 (27)	Moderate ⨁⨁⨁◯ Due to the risk of bias and inconsistency	We are moderately confident in the effect estimate: the true effect is likely to be close to the estimate of the effect, but there is a possibility that it is different
72 h post	123 (10)	Moderate ⨁⨁⨁◯ Due to the risk of bias and inconsistency	We are moderately confident in the effect estimate: the true effect is likely to be close to the estimate of the effect, but there is a possibility that it is different

*n* denotes the number of participants and *k* denotes the number of studies.

The certainty of evidence for glutathione measurements was consistently rated as high across all time points evaluated. Immediately post‐exercise (378 participants, 32 studies), at 30 min to 2 h post‐exercise (165 participants, 13 studies) and at 48 h post‐exercise (232 participants, 13 studies) the evidence demonstrated a high level of confidence in the effect estimates. This indicates that the true effect is highly likely to be close to the observed estimates across all these time points.

The certainty of evidence for F_2_‐isoprostanes varied by time point. Immediately post‐exercise (511 participants, 37 studies), the evidence was rated as moderate, suggesting reasonable confidence in the effect estimate with some possibility of deviation. In contrast, measurements taken 30 min to 2 h post‐exercise (181 participants, 13 studies) were associated with a low certainty rating, reflecting limited confidence and a potential for substantial differences from the true effect.

The certainty of evidence for protein carbonyls varied considerably across time points. Immediately post‐exercise (528 participants, 40 studies), the certainty was low. At 30 min to 2 h post‐exercise (186 participants, 16 studies), the certainty improved to moderate. Measurements at 24 h post‐exercise (283 participants, 24 studies) had low certainty again, indicating a limited level of confidence in the effect estimate. In contrast, at both 48 h (393 participants, 27 studies) and 72 h post‐exercise (123 participants, 10 studies), the certainty was again moderate.

## 4 DISCUSSION

### 4.1 Main findings

**FIGURE 16:**
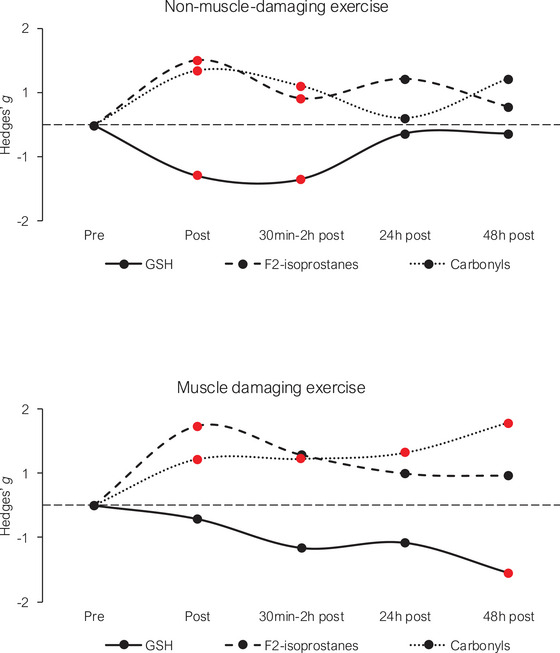
Time‐course dynamics of effect sizes for glutathione (GSH), F_2_‐isoprostanes and protein carbonyls following non‐muscle‐damaging exercise (upper panel) and muscle‐damaging exercise (lower panel) over a 48‐h post‐exercise period. Red circles denote time points with statistically significant effects.

The present systematic review and meta‐analysis produced three primary findings: (i) acute exercise induces an increase in oxidative stress levels, with effect sizes – calculated as Hedges’ *g* – ranging from 0.41 to 1.33, depending on the biomarker measured, the time point of assessment, the biological specimen used and the exercise protocol applied; (ii) subgroup analyses revealed that, in general, the optimal time point to assess oxidative stress following acute exercise appears to be immediately post‐exercise for non‐muscle‐damaging exercise protocols and 48 h post‐exercise for muscle‐damaging exercise protocols; (iii) further subgroup analyses indicated that erythrocytes are the preferred specimen for glutathione measurement immediately post‐exercise, whilst plasma and urine are almost equally suitable for F_2_‐isoprostane measurement at this time point. Exploratory specimen‐restricted sensitivity analyses across biomarkers and time points were broadly consistent with the primary findings (i.e., effect estimates and inferred peak timing) but imprecise, reflecting a small number of studies and residual heterogeneity. Thus, we interpret subset results cautiously. Figure 16 illustrates the time‐course dynamics of effect sizes for the three biomarkers in response to non‐muscle‐damaging exercise (upper panel) and muscle‐damaging exercise (lower panel) up to 48 h post‐exercise, whilst Table 2 provides the quantitative details.

**TABLE 2:** Effect sizes (Hedges’ *g* / CLES) for the significant findings of the meta‐analysis.

Biomarker	Immediately post	30 min–2 h post	24 h post	48 h post
Glutathione (*k* = 41)				
All studies	−0.70/68.97% (*k* = 32)	−0.81/71.66% (*k* = 13)		−0.98/75.58% (*k* = 13)
Non‐muscle‐damaging	−0.79/71.18% (*k* = 27)	−0.85/72.61% (*k* = 9)		
Muscle‐damaging				−1.05/77.11% (*k* = 12)
F_2_‐isoprostanes (*k* = 40)				
All studies	1.01/76.24% (*k* = 37)	0.46/62.75% (*k* = 13)		
Non‐muscle‐damaging	1.00/76.02 % (*k* = 34)	0.41/61.41% (*k* = 11)		
Muscle‐damaging				
Protein carbonyls (*k* = 52)				
All studies	0.79/71.18% (*k* = 40)	0.65/67.71% (*k* = 16)	0.64/67.46% (*k* = 24)	1.17/79.60% (*k* = 27)
Non‐muscle‐damaging	0.84/72.37% (*k* = 27)	0.60/66.43% (*k* = 10)		
Muscle‐damaging	0.72/69.47% (*k* = 13)	0.73 /69.71% (*k* = 6)	0.82/71.9% (*k* = 18)	1.28/81.73% (*k* = 21) **72h**: 1.33/82.65% (*k* = 10)

CLES, common language effect size; *k* denotes the number of studies.

The differential redox dynamics between exercise types likely reflects distinct underlying sources of oxidative stress: in non‐muscle‐damaging exercise, ROS generation is transient, returns to baseline relatively quickly (i.e., few hours), and is primarily metabolic in origin, with NADPH oxidases playing a central role (Specht et al., 2021); by contrast, the delayed and sustained increase in oxidative stress observed following muscle‐damaging exercise appears to be predominantly inflammatory in origin, driven by immune cell infiltration and regenerative processes (Nikolaidis et al., 2008). It should be noted, however, that for time points where non‐significant findings were reported, the number of available studies was typically small (ranging from 2 to 6 studies), such as for muscle‐damaging exercise immediately post‐exercise or 30 min to 2 h post‐exercise, and for non‐muscle‐damaging exercise at 48 h post‐exercise. We qualify our conclusions by noting that we did not account for exercise intensity or the occurrence of fatigue. These factors likely modulate the magnitude of early and late redox responses, even if the timing of peak responses remains biomarker‐ and protocol‐specific.

### 4.2 Interpretation of findings

Although standardised mean differences (SMD; Hedges’ *g*) were used throughout the analyses to allow comparisons across studies using different units and methods, interpreting these values in real‐world biological terms is always a matter of debate. In behavioural sciences, Cohen's thresholds (i.e., 0.2 small, 0.5 medium, 0.8 large) are often used as general benchmarks, but they may not directly apply to other fields. For instance, the *g* = 0.84 observed for protein carbonyls immediately after non‐muscle‐damaging exercise theoretically suggests a substantial biological perturbation in protein oxidation levels. Yet, we should be cautious about this conclusion, especially considering that no redox biomarker has ever been quantitatively linked to meaningful physiological or clinical outcomes (Lichtenberg et al., 2023; Margaritelis, Cobley, et al., 2016; Nikolaidis et al., 2020). To provide another tangible example, the *g* = 1.00 for the same exercise type in F_2_‐isoprostanes immediately post‐exercise, corresponds roughly to an increase of ∼1 standard deviation compared to baseline levels. Thus, if the baseline urine F_2_‐isoprostanes concentration was, for instance, 300 pg/mg creatinine with a standard deviation of 100 pg/mg, a *g* equal to 1.00 would correspond to an absolute increase of ∼100 pg/mg creatinine (∼33%). In practical terms, this would represent a significant shift in oxidative stress levels, potentially relevant for signalling and adaptation processes, though the exact causal links remain vague. Of course, this oversimplified example assumes that the post‐exercise variance is roughly the same as the baseline (pre‐exercise) variance. However, biological data sets rarely meet this assumption, as they typically exhibit a positive mean‐variance relationship, where higher mean values are associated with higher variances (a pattern known as heteroscedasticity) (Steele et al., 2023). Yet, the mean‐variance modelling concept is beyond the scope of the present work, and the example provided above was intentionally simplified for illustrative purposes.

Another more intuitively comprehensible statistic is the common language effect size (CLES), also known as the probability of superiority (Grissom, 1994; McGraw & Wong, 1992). The CLES expresses the probability that a randomly selected individual from one group will have a higher observed value than a randomly selected individual from another group; in a within‐subject (paired) design, it corresponds to the probability that a randomly selected individual's change score after a treatment will be greater than zero (Caldwell & Vigotsky, 2020). The CLES can be calculated using the equation CLES = Φ(g/√2), where Φ denotes the cumulative distribution function of the standard normal distribution. Accordingly, the CLES is 0.50 (or 50%) when *g* = 0 and approaches 1 (or 100%) as *g* increases (Cooper et al., 2019). For practical interpretation, Table 2 provides the corresponding CLES values for all significant effect sizes identified in the present meta‐analyses.

In interpreting the remaining findings of the present meta‐analysis, several points deserve special consideration. First, although the overall quality of the included studies was generally moderate‐to‐high, significant heterogeneity was observed across almost all analyses (*I* ^2^ values often exceeding 60%, whilst τ^2^ values were also large), suggesting that factors beyond sampling error contributed to the observed variability. Such heterogeneity may reflect differences in exercise protocols, sample handling, biomarker assays, participant characteristics and study designs, among others. Importantly, prediction intervals, illustrated as a thick black line below the diamond representing the overall effect in the forest plots, were wide in most analyses, sometimes even crossing zero. This highlights that future studies could still observe effects ranging from negative or trivial to large, depending on study‐specific conditions (Borenstein et al., 2021). Second, although publication bias was generally low for glutathione, some small‐study effects were detected for F_2_‐isoprostanes and protein carbonyls immediately post‐exercise, as indicated by asymmetrical funnel plots and significant Egger's tests. This suggests that the magnitude of oxidative stress responses reported in the literature, particularly at early time points post‐exercise, might be slightly overestimated. Yet, the overall robustness of the findings was supported by the non‐significant Egger's tests at later time points and our sensitivity analyses (data not shown). Third, the GRADE assessment provided important insights into the quality of evidence across different time points for the oxidative stress biomarkers. In particular, glutathione had consistently high certainty across all time points, suggesting it is a reliable marker for oxidative stress assessment. On the other hand, the certainty of evidence for F_2_‐isoprostanes and protein carbonyls was more variable and questionable at earlier and intermediate time points, but not for delayed time points (i.e., 48 and 72 h post‐exercise) after muscle‐damaging exercise. Fourth, according to the subgroup and sensitivity analyses based on the biological specimen, it is evident that, due to their minimal invasiveness, redox measurements in blood plasma, erythrocytes and urine are by far the most common in human exercise studies (Chatzinikolaou et al., 2024). In contrast, skeletal muscle measurements are scarce; however, they are of critical importance, given that redox signalling processes regulating exercise responses and adaptations exhibit a highly compartmentalised nature within skeletal muscle cells (Henriquez‐Olguin et al., 2023; Margaritelis et al., 2020b). Yet, it is important to keep in mind that compartment‐specific kinetics, encompassing generation, diffusion across membranes and clearance, can introduce appearance delays and thereby influence observed biomarker time courses across specimens and their interpretation (Halliwell & Lee, 2010). Fifth, non‐damaging exercise elicits a short‐lived oxidant burst driven by transient increases in NADPH oxidase activity, shear‐stress‐related xanthine oxidase and mitochondrial electron ‘leak’ post‐exercise, which is rapidly buffered by existing antioxidant capacity and early Nrf2‐dependent responses. By contrast, muscle‐damaging exercise initiates a secondary inflammatory phase with neutrophil/macrophage infiltration, NOX (NADPH oxidases)/MPO (myeloperoxidase)‐derived oxidants, lingering mitochondrial perturbation, and iron‐catalysed reactions during remodelling, shifting and broadening biomarker peaks into the 24–72 h window. Differences in compartmentalisation (intra‐ vs. extracellular), release/clearance kinetics and assay specificity further shape the apparent timing across markers.

### 4.3 Implications of findings

The findings of the present meta‐analysis may have important implications across five key areas. First, reduced waste of resources by limiting oxidative stress measurements to the most informative time points. Currently, many studies monitor oxidative stress over extended periods without a clear experimental rationale, leading to potential resource inefficiencies. Second, employing such methodological standardisation strategies will facilitate the production of more comparable data across studies. This standardisation is crucial given other methodological inconsistencies that persist in the relevant literature, such as the wide array of analytical techniques (Murphy et al., 2022). Third, the effect sizes reported in the present meta‐analysis can assist future studies in accurately calculating sample sizes. This is of major importance in the redox biology field, where, in most studies, the sample size is either not justified, frequently leading to underpowered studies, or is calculated using default effect sizes (i.e., arbitrary benchmarks) derived from other fields (e.g., Cohen's *d*; Cohen, 2013). In the same context, no oxidative stress biomarker change has yet been quantitatively linked to a physiological (e.g., increased performance) or clinical outcome (e.g., muscle atrophy) in order to be used alternatively as the smallest effect size of interest in sample size calculations (Hislop et al., 2014). Fourth, knowing the optimal time points for evaluating exercise‐induced oxidative stress allows for better timing of other redox interventions along with exercise. In particular, aligning the redox ‘kinetics’ of an intervention with exercise‐induced oxidative stress changes may enhance its effectiveness (see Figure 14 in Nikolaidis et al. (2015) for an example with acute antioxidant supplementation). Fifth, the quantitative data reported can feed computational redox models and mathematical simulations, whose usefulness is increasingly acknowledged in redox biology (Pillay & Rohwer, 2024; Sousa et al., 2022).

### 4.4 Meta‐analysis as a research synthesis method in redox biology

Meta‐analysis, a term introduced by Glass (1976), but conceptually older (e.g., Pearson's work; Pearson, 1904), is now central to research synthesis (for a historical overview, see Chapter 1 in Egger et al., 2022). It is now clear that we are living in an era dominated by research synthesis. We will not present a timeline histogram from PubMed illustrating the increasing annual frequency of publications containing the term ‘meta‐analysis’, because this trend actually mirrors the overall rise in scientific output across all disciplines. However, certain qualitative indicators underscore its importance, such as the reliance of guidelines and position statements on meta‐analytic data. Despite these facts, systematic reviews and meta‐analyses remain underutilised in the field of redox biology [e.g., exercise and/or nutrition (Martinez‐Negrin et al., 2022; Tan et al., 2023; Tryfidou et al., 2020), diseases (Causer et al., 2020; van ’t Erve et al., 2017; Wu et al., 2025)], even though substantial data are now available. At the same time, meta‐analyses hold potential for bridging disparate perspectives across disciplines and shaping a unifying framework to better understand the role of redox processes in biology (Nikolaidis & Margaritelis, 2018).

The advantages of meta‐analysis are well‐documented and include greater statistical power, more precise effect estimates, and narrower confidence intervals than individual studies (Borenstein et al., 2021), which is crucial given the small sample sizes typical in exercise and translational redox biology research. Meta‐analyses also reduce subjectivity compared to narrative reviews by quantitatively weighting studies by precision, avoiding the common questionable approach used in narrative reviews, which dichotomously classifies results as statistically ‘significant’ or ‘non‐significant’ based on their *P*‐values relative to a chosen α level, and draws conclusions by counting the number of studies in each category. The latter practice essentially mimics the ‘vote counting’ approach in meta‐analysis, which is strongly discouraged (Chapter 33 in Borenstein et al., 2021). The vote‐counting approach is flawed for two main reasons. First, it treats non‐significant *P*‐values as evidence for the absence of an effect, a misinterpretation that has been widely criticised (Altman & Bland, 1995). Second, the typically low statistical power of individual studies, coupled with random variability, may work strange wonders (Senn, 2021).

Despite the aforementioned advantages, meta‐analyses are not without critique (see Chapter 48 in Borenstein et al. (2021) for a detailed presentation of arguments and counterarguments). Beyond technical challenges like publication bias and study quality, two issues are especially relevant in redox biology. First, inclusion/exclusion criteria are inherently subjective: a curated collection of studies with shared characteristics is like curating a ‘best dishes’ cookbook, where apparently objective endpoints (e.g., creativity, techniques and presentation) depend on subjective choices that reflect the authors’ personal preferences or biases (e.g., a minimum rating on specific culinary websites or endorsement from renowned chefs). Likewise, in a meta‐analysis, the objective outputs may be influenced by subjective judgments for study inclusion, such as the analytical techniques applied or the specimen used. Thus, transparent, rigorous and reasonable criteria are essential. Second, the epistemological rationale underpinning the research question matters critically. Weak or oversimplified theoretical frameworks may introduce biases, whether positive or negative, towards a particular (non)causal relationship. For example, studies that employ ‘antioxidant agents’ as therapeutic aids in conditions characterised by oxidative stress most often (if not always) yield negative results, leading to broad dismissals of antioxidants as ineffective (Margaritelis et al., 2024). However, this approach reflects a theoretical oversimplification, as it assumes a direct causal role for reactive species in the pathophysiology of diseases, a premise increasingly called into question (Halliwell, 2024).

### 4.5 Redox biomarkers and their utility in translational research

Despite substantial progress in redox biology, the translational application of oxidative stress biomarkers remains limited (Daiber et al., 2021; Frijhoff et al., 2015; Margaritelis, Cobley, et al., 2016; van ’t Erve et al., 2017; Veskoukis et al., 2020; Weber et al., 2017). One major challenge is the lack of standardised reference intervals across different laboratories and techniques, which complicates the clinical interpretation and broader applicability of these biomarkers (Aravapally et al., 2025; Cobley et al., 2017; Mas‐Bargues et al., 2022; Murphy et al., 2022). In our meta‐analysis, standardised mean differences were used precisely because absolute concentrations of redox biomarkers were measured using heterogeneous methods across studies. This methodological diversity impedes the establishment of universal diagnostic or prognostic thresholds necessary for clinical translation. Moreover, oxidative stress biomarker studies often suffer from biological sampling issues. For instance, biomarkers are frequently measured in tissues or fluids where they are either unstable or biologically irrelevant. For example, glutathione, a predominantly intracellular antioxidant (Berndt & Lillig, 2017; Deponte, 2017), is sometimes assessed in plasma or urine, despite evidence against this approach due to the lack of a biologically plausible mechanism of action in these matrices. Such practices raise concerns about biological plausibility and hinder the interpretation of findings in a mechanistic or translational context.

Beyond reference‐interval heterogeneity, assay‐ and pre‐analytical variability across all three biomarkers of the present meta‐analysis further limit translation. For F_2_‐isoprostanes, some immunoassays exhibit specimen‐dependent bias and cross‐reactivity with prostanoid species, whereas methods like GC/LC‐MS offer superior specificity and should be preferred (Milne et al., 2015; Nikolaidis et al., 2011). This is of major importance to distinguish the origins of acute lipid peroxidation, namely, the chemical (i.e., free radical‐mediated arachidonic acid oxidation) from enzymatic (e.g., prostaglandin‐endoperoxide synthase‐mediated) lipid peroxidation, with only the former being consistent with oxidative stress (Van'T Erve et al., 2016). For glutathione, valid assessment requires specimen‐appropriate sampling (e.g., erythrocytes or skeletal muscle), immediate derivatisation/stabilisation (e.g., with *N*‐ethylmaleimide), strict cold‐chain handling, and avoidance of freeze–thaw, as isolated GSH is highly susceptible to *ex vivo* artifacts (Giustarini et al., 2013). For protein carbonyls, methods such as 2,4‐dinitrophenylhydrazine spectrophotometry, enzyme‐linked immunosorbent assay and immunochemical detection differ in calibration, protein normalisation and susceptibility to interferents, yielding divergent absolute values and variances (Weber et al., 2015). These methodological differences can shift effect estimates and heterogeneity. Nonetheless, we adopted an inclusive approach, retaining all eligible studies irrespective of analytical technique, because kit‐based assays remain widely used in the field and restricting analyses to gold‐standard methods, which are rarely available in translational settings, would leave too few studies per biomarker and time point for robust random‐effects meta‐analysis. This pragmatic choice preserves power and increases external validity, although we clearly acknowledge this validity‐ and reliability‐related experimental limitation.

Another critical limitation regarding the utility of redox biomarkers in translational research is the under‐representation of specific populations, such as females. As highlighted in a recent systematic review (James et al., 2023), women remain significantly under‐represented in human physiology studies, including oxidative stress research and in clinical research in general (Bierer et al., 2022; Daitch et al., 2022). In the context of the present meta‐analysis, across the 103 included original studies, only 13 enrolled female subjects. Moreover, of 1418 total participants, only 124 were female (8.7%). Sex‐stratified subgroup analyses were not feasible because, within each biomarker and time point, the number of studies with female data was well below our a priori threshold, yielding insufficient power and unstable estimates. Where possible, exploratory sensitivity checks did not materially alter effect sizes or inferred peak timing, but these analyses again remained underpowered and should be interpreted cautiously. Given known sex‐based differences in redox regulation, this bias restricts the generalisability of current findings and warrants correction in future investigations. Emerging evidence indicates that sex hormones, particularly oestrogens, may positively influence mitochondrial dynamics (e.g., by expressing mitochondria‐localised nuclear genes, such as *DRP1* and *mitofusin*) and antioxidant defenses (Di Florio et al., 2020). However, findings are context‐dependent and do not definitively support a general claim of superiority in females. We therefore avoid categorical language and emphasise the need for sex‐aware study designs. Additionally, it has been emphasised that redox responses are profoundly sex‐dependent, with implications for disease susceptibility and progression (Straface et al., 2017). Ignoring these biological differences may not only limit the applicability of redox biomarkers but also obscure important mechanistic insights into sex‐specific vulnerabilities and adaptive responses, for instance, in response to an exercise protocol. Future research must therefore prioritise the balanced inclusion of both sexes and adopt analytical frameworks capable of dissecting sex‐specific redox signatures.

For oxidative stress biomarkers to be more widely used in translational research, several steps are necessary: (i) harmonisation of measurement protocols and calibration across laboratories; (ii) careful selection of biologically relevant tissues and fluids for sampling; (iii) establishment of reference intervals that account for age, sex, fitness level and other biological variables to quantitatively define eustress and distress (Margaritelis & Nikolaidis, 2025); and (iv) validation of biomarker changes against meaningful clinical or functional outcomes (e.g., exercise adaptations or disease progression).

### 4.6 Next steps: expanding insights into clinical and exercise redox biology

To the best of our knowledge, no single biomarker fully satisfies all four criteria mentioned above (i.e., harmonisation, biologically relevant matrix, reference intervals, validation) across settings; yet, a few currently used candidates, such as F_2_‐isoprostanes (8‐iso‐PGF2α) and 8‐oxo‐7,8‐dihydro‐2′‐deoxyguanosine (8‐oxodG) measured via LC‐MS/MS or HPLC with electrochemical detection, are comparatively well‐validated and come closest to these requirements (Chiorcea‐Paquim, 2022; Nikolaidis et al., 2011). However, future original research and meta‐analyses could prioritise the direct quantification of acute exercise‐induced changes in specific reactive species (e.g., hydrogen peroxide or nitric oxide), redox‐sensitive transcription factors (e.g., Nrf2 or NF‐κB) or redox‐related signalling pathways (e.g., the Nrf2–Keap1–catalase pathway; Gallego‐Selles et al., 2020). Moving beyond traditional oxidative stress biomarkers, these molecules offer direct mechanistic insights into how redox perturbations orchestrate molecular adaptations to exercise. Advances in genetically encoded redox biosensors (Kritsiligkou et al., 2023; Pedre, 2024) now allow real‐time, compartment‐specific measurements of reactive species and should be increasingly incorporated into exercise research protocols.

In addition, whilst the current meta‐analysis focused on acute exercise responses, future work should investigate how chronic exercise training modulates resting oxidative stress levels. Understanding the kinetics and magnitude of resting redox adaptations (e.g., antioxidant capacity, oxidative stress biomarkers) after weeks or months of structured and supervised training would provide critical information about redox remodelling in health and disease contexts (Caporossi & Dimauro, 2024; Henriquez‐Olguin et al., 2020; Margaritelis et al., 2020b).

Furthermore, given the highly compartmentalised nature of redox signalling, future studies should systematically assess redox changes across multiple tissues, such as plasma (Veskoukis et al., 2018), erythrocytes (Chatzinikolaou et al., 2024), skeletal muscle (Henríquez‐Olguín et al., 2019), extracellular vesicles (Lisi et al., 2023) and even from specific organelles like mitochondria (Sanz‐Ros et al., 2023). Such compartment‐specific profiling could reveal important patterns missed by systemic measurements alone and better link redox responses to functional outcomes (e.g., exercise fatigue). Thus, there is a great need for head‐to‐head paired designs with standardised timing, harmonised analytical methods and appropriate normalisation (e.g., creatinine for urine, haemoglobin for erythrocytes) to evaluate whether non‐invasive sampling approaches offer comparable sensitivity and specificity to biopsy measurements, with clear implications for ethics and cost.

Finally, redox biology research would benefit from the integration of quantitative data into computational models and simulations (Antunes & Brito, 2017; Pillay & Rohwer, 2024; Sousa et al., 2022). Building predictive models based on time‐course changes in oxidative stress biomarkers and their upstream/downstream pathways could help forecast physiological responses to different types of exercise (endurance vs. resistance exercise), facilitating the design of optimised training interventions and personalised strategies for health and performance (Margaritelis, 2023).

## 5 Limitations

This systematic review and meta‐analysis has a number of limitations. First, the three outcome variables analysed are generic biomarkers of oxidative stress, which offer limited mechanistic insight into the redox regulation of exercise biology (i.e., responses or adaptations). Initially, we aimed to include hydrogen peroxide as the most widely studied reactive species with signalling properties (Henriquez‐Olguin et al., 2020; Pedre, 2024; Veal & Kritsiligkou, 2024), as well as a redox‐regulated transcription factor that relates to exercise, such as NF‐κB or Nrf2 (Gallego‐Selles et al., 2022; Martinez‐Canton et al., 2024; Ostrom et al., 2021). Given that our meta‐analysis focused on human studies, the available data for these molecules were too sparse and heterogeneous to support a meaningful quantitative synthesis. It would nonetheless be valuable to understand how these molecules fluctuate within skeletal muscle during and after exercise. Second, no restrictions were placed on the type of acute exercise included in our analysis, resulting in a highly heterogeneous pool of studies with varying protocols in terms of exercise volume, duration, intensity, fitness status, fibre type/phenotype and fatigue occurrence, all of which may partially modulate biomarker kinetics and effect sizes (Alessio et al., 2000; Quindry et al., 2003). This lack of standardisation is an inherent challenge in the sports science literature, compounded by other common methodological limitations, such as the inability to employ double‐ or single‐blind designs for training protocols (i.e., participants are aware of their activity) or to match training volume or intensity between different protocols strictly. Third, in line with the previous limitation, we classified exercise protocols only by their muscle‐damaging or non‐damaging component. We labelled an acute exercise session as muscle‐damaging under three conditions: (i) if explicitly stated by the authors in the original study (e.g., eccentric contractions on an isokinetic dynamometer); (ii) when the exercise type is widely recognised to include a strong eccentric component (e.g., downhill running); or (iii) if the original study assessed a biochemical or physiological marker of muscle damage, such as creatine kinase or delayed onset muscle soreness (Peake et al., 2017). Fourth, we included all eligible studies regardless of analytical technique, partially introducing methodological variability that could inflate or attenuate pooled effect estimates; yet, using standardised mean differences (i.e., Hedges’ *g*) moderately mitigates this issue in the sense of harmonising scale differences. Lastly, since most studies used blood plasma, serum or erythrocytes as sample types, we were unable to perform a subgroup analysis across different tissues for all time points. In particular, only a few studies employed human skeletal muscle biopsies or urine samples, which limited the potential for tissue‐specific sub‐analyses to specific cases.

## 6 Conclusion

This systematic review and meta‐analysis demonstrate that the optimal timing to assess oxidative stress following acute exercise in humans is both biomarker‐ and protocol‐specific. Non‐muscle‐damaging exercise elicits early redox responses within 2 h, whilst muscle‐damaging protocols (i.e., eccentric exercise) produce delayed peaks, typically at 48–72 h. The consistency of the findings across a wide range of exercise modalities, biological specimens and study designs reinforces the notion that oxidative stress is a fundamental, time‐sensitive component of the acute exercise response. Amongst the biomarkers assessed, glutathione provided the highest certainty of evidence and the lowest risk of bias, whereas F_2_‐isoprostanes and protein carbonyls were affected by small‐study effects and methodological heterogeneity. By providing research‐informed timing recommendations for commonly assessed redox biomarkers, this study offers a basis for more standardised and informative experimental designs, improved sample size estimation and ultimately more meaningful links between redox biology and functional outcomes in exercise physiology and sports nutrition.

## AUTHOR CONTRIBUTIONS

*Conception or design of the work*: Anastasios A. Theodorou, Vassilis Paschalis, Ioannis S. Vrabas, Antonios Kyparos, Athanasios Z. Jamurtas, Ioannis G. Fatouros, Michalis G. Nikolaidis and Nikos V. Margaritelis. *Acquisition, analysis or interpretation of data for the work*: Chrysovalantis Stachteas, Nikolaos Georgogiannis, George G. Nastos, Panagiotis N. Chatzinikolaou, Petros C. Dinas and Nikos V. Margaritelis. All authors have contributed to drafting the work or revising it critically for important intellectual content. All authors approved the final version of the manuscript; agree to be accountable for all aspects of the work in ensuring that questions related to the accuracy or integrity of any part of the work are appropriately investigated and resolved; and all persons designated as authors qualify for authorship, and all those who qualify for authorship are listed.

## CONFLICT OF INTEREST

The authors have no competing interests to declare that are relevant to the content of this article.

## FUNDING INFORMATION

This research did not receive any specific grant from funding agencies in the public, commercial, or not‐for‐profit sectors.

## Supporting information



Supplementary File 1. Tables S1–S4, Figures S1–S7.

Supplementary File 2. Summary of all meta‐analyses and subgroup analyses.

Supplementary File 3. Grade analysis.

## Data Availability

All raw data from the included studies (i.e., sample sizes, means, and standard deviations) for all biomarkers and time points, as well as the respective effect sizes (i.e., Hedges’ *g*) of each study, are available upon request. In addition, Supplementary File 2 includes a comprehensive summary of all meta‐analyses and subgroup analyses performed.
