# Supplementary material for: Optimal timing to assess exercise‐induced oxidative stress: A systematic review and meta‐analysis
Source: Exp Physiol. 2025 Nov 29:10.1113/EP092963. Online ahead of print. doi: 10.1113/EP092963 (PMC13394821; doi:10.1113/EP092963)

**Optimal timing to assess exercise-induced oxidative stress: a systematic review and meta-analysis**

Chrysovalantis Stachteas^1,#^, Nikolaos Georgogiannis^1,#^, George G. Nastos^1,#^, Panagiotis N. Chatzinikolaou^1^, Petros C. Dinas^2^, Anastasios A. Theodorou^3^, Vassilis Paschalis^4^, Ioannis S. Vrabas^1^, Antonios Kyparos^1^, Athanasios Z. Jamurtas^2^, Ioannis G. Fatouros^2^, Michalis G. Nikolaidis^1^, Nikos V. Margaritelis^1,^*

^1^ Department of Physical Education and Sports Science at Serres, Aristotle University of Thessaloniki, Serres, Greece

^2^ School of Physical Education, Sport Science and Dietetics, University of Thessaly, Trikala, Greece

^3^ Department of Life Sciences, School of Sciences, European University Cyprus, Nicosia, Cyprus

^4^ School of Physical Education and Sport Science, National and Kapodistrian University of Athens, Athens, Greece

^#^ These authors contributed equally

***Corresponding author**

Nikos V. Margaritelis, PhD

Department of Physical Education and Sports Science at Serres

Aristotle University of Thessaloniki

Agios Ioannis 62122, Serres

Greece

Tel: +30 2310991040

nvmargar@auth.gr

ORCiD: 0000-0001-5119-427X

**SUPPLEMENTARY FILE 1**

Table of Contents

[Table S1. Keyword search algorithms 2](#_Toc196833219)

[Table S2. PRISMA 2020 Checklist 4](#_Toc196833220)

[Table S3. Eligible study characteristics 6](#_Toc196833221)

[Table S4. Publication bias assessment 14](#_Toc196833222)

[Figure S1. PRISMA 2020 flow diagram for glutathione 17](#_Toc196833223)

[Figure S2. PRISMA 2020 flow diagram for F_2_-isoprostanes 18](#_Toc196833224)

[Figure S3. PRISMA 2020 flow diagram for protein carbonyls 19](#_Toc196833225)

[Figure S4. Risk of bias assessment with ROB2 traffic light plot 20](#_Toc196833226)

[Figure S5. Risk of bias assessment with ROBINS-I traffic light plot 21](#_Toc196833227)

[Figure S6. Subgroup analysis based on the biological specimen (i.e., erythrocyte vs. skeletal muscle) for GSH immediately post-exercise 22](#_Toc196833228)

[Figure S7. Subgroup analysis based on the biological specimen (i.e., plasma vs. urine) for F_2_-isoprostanes immediately post-exercise 23](#_Toc196833229)

# Table S1. Keyword search algorithms

| **Biomarker** | **Algorithm** |
| --- | --- |
| Glutathione | PubMed |
|  | (((((Glutathione[Title/Abstract]) OR (gsh[Title/Abstract]) OR (GSH[Title/Abstract]) OR (γ-L-Glutamyl-L-cysteinylglycine[Title/Abstract]) AND ((physical activity[Title/Abstract]) OR (intervention[Title/Abstract]) OR (running[Title/Abstract]) OR (marathon[Title/Abstract]) OR (race[Title/Abstract]) OR (exercise[Title/Abstract]) OR (training[Title/Abstract]) OR (aerobic training[Title/Abstract]) OR (fitness[Title/Abstract]) OR (strength exercise[Title/Abstract]) OR (resistance training[Title/Abstract]) OR (acute exercise[Title/Abstract]) OR (sports[Title/Abstract]) OR (game[Title/Abstract]) OR (match[Title/Abstract]) OR (plyometric training[Title/Abstract]))) |
|  | EMBASE |
|  | 'glutathione'/exp OR 'glutathione':ti,ab OR 'gsh':ti,ab OR 'γ-l-glutamyl-l-cysteinylglycine':ti,ab AND 'physical activity'/exp OR 'physical activity':ti,ab OR intervention:ti,ab OR running:ti,ab OR marathon:ti,ab OR race:ti,ab OR exercise:ti,ab OR training:ti,ab OR 'aerobic training':ti,ab OR fitness:ti,ab OR 'strength exercise':ti,ab OR 'resistance training':ti,ab OR 'acute exercise':ti,ab OR sports:ti,ab OR game:ti,ab OR match:ti,ab OR 'plyometric training':ti,ab |
|  | Scopus |
|  | TITLE-ABS("glutathione") OR TITLE-ABS("gsh") OR TITLE-ABS("GSH") OR TITLE-ABS("γ-L-Glutamyl-L-cysteinylglycine") AND TITLE-ABS("physical activity") OR TITLE-ABS("intervention") OR TITLE-ABS("running") OR TITLE-ABS("marathon") OR TITLE-ABS("race") OR TITLE-ABS("exercise") OR TITLE-ABS("training") OR TITLE-ABS("aerobic training") OR TITLE-ABS("fitness") OR TITLE-ABS("strength exercise") OR TITLE-ABS("resistance training") OR TITLE-ABS("acute exercise") OR TITLE-ABS("sports") OR TITLE-ABS("game") OR TITLE-ABS("match") OR TITLE-ABS("plyometric training") |
|  |  |
| F_2_-isoprostanes | PubMed |
|  | (((((isoprostanes[Title/Abstract]) OR (F2 isoprostanes[Title/Abstract]) OR (IsoPs[Title/Abstract]) OR (F2-IsoPS[Title/Abstract]) OR (PGF2a[Title/Abstract]) OR (15-F2t-IsoP[Title/Abstract]) OR (8-iso-PGF2a[Title/Abstract]) OR (8-iso.PGF2a[Title/Abstract])) AND ((physical activity intervention[Title/Abstract]) OR (intervention[Title/Abstract]) OR (exercise[Title/Abstract]) OR (physical activity[Title/Abstract]) OR (training[Title/Abstract]) OR (aerobic training[Title/Abstract]) OR (fitness[Title/Abstract]) OR (strength exercise[Title/Abstract]) OR (resistance training[Title/Abstract]) OR (acute exercise[Title/Abstract]) OR (plyometric training[Title/Abstract]))) |
|  | EMBASE |
|  | 'isoprostane'/exp OR 'f2 isoprostane':ti,ab OR 'isoprostanes':ti,ab OR 'isops':ti,ab OR 'f2-isops':ti,ab OR 'pgf2a':ti,ab OR '15-f2t-isop':ti,ab OR '8-iso-pgf2a':ti,ab OR '8-iso.pgf2a':ti,ab AND 'exercise'/exp OR 'physical activity'/exp OR 'physical activity intervention':ti,ab OR intervention:ti,ab OR exercise:ti,ab OR 'physical activity':ti,ab OR training:ti,ab OR 'aerobic training':ti,ab OR fitness:ti,ab OR 'strength exercise':ti,ab OR 'resistance training':ti,ab OR 'acute exercise':ti,ab OR 'plyometric training':ti,ab |
|  | Scopus |
|  | TITLE-ABS("isoprostanes") OR TITLE-ABS("f2 isoprostanes") OR TITLE-ABS("isops") OR TITLE-ABS("f2-isops") OR TITLE-ABS("pgf2a") OR TITLE-ABS("15-f2t-isop") OR TITLE-ABS("8-iso-pgf2a") OR TITLE-ABS("8-iso.pgf2a") AND TITLE-ABS("physical activity intervention") OR TITLE-ABS("intervention") OR TITLE-ABS("exercise") OR TITLE-ABS("physical activity") OR TITLE-ABS("training") OR TITLE-ABS("aerobic training") OR TITLE-ABS("fitness") OR TITLE-ABS("strength exercise") OR TITLE-ABS("resistance training") OR TITLE-ABS("acute exercise") OR TITLE-ABS("plyometric training")” |
|  |  |
| Protein carbonyls | PubMed |
|  | (((((acute exercise[Title/Abstract]) OR (exercise[Title/Abstract]) OR (physical exercise[Title/Abstract]) OR (training[Title/Abstract]) OR (acute training[Title/Abstract]) OR (post-exercise[Title/Abstract])) AND ((protein oxidation[Title/Abstract]) OR (protein carbonyls[Title/Abstract]) OR (carbonyls[Title/Abstract]) OR (protein carbonylation[Title/Abstract]) OR (carbonylation[Title/Abstract]) OR (oxidative stress[Title/Abstract]))) |
|  | EMBASE |
|  | 'exercise'/exp OR 'physical exercise':ti,ab OR 'acute exercise':ti,ab OR 'training':ti,ab OR 'acute training':ti,ab OR 'post-exercise':ti,ab AND 'oxidative stress'/exp OR 'protein oxidation':ti,ab OR 'protein carbonyls':ti,ab OR 'carbonyls':ti,ab OR 'protein carbonylation':ti,ab OR 'carbonylation':ti,ab OR 'oxidative stress':ti,ab |
|  | Scopus |
|  | TITLE-ABS("acute exercise") OR TITLE-ABS("exercise") OR TITLE-ABS("physical exercise") OR TITLE-ABS("training") OR TITLE-ABS("acute training") OR TITLE-ABS("post-exercise") AND TITLE-ABS("protein oxidation") OR TITLE-ABS("protein carbonyls") OR TITLE-ABS("carbonyls") OR TITLE-ABS("protein carbonylation") OR TITLE-ABS("carbonylation") OR TITLE-ABS("oxidative stress") |

# Table S2. PRISMA 2020 Checklist

| **Section and Topic** | **Item #** | **Checklist item** | **Location where item is reported** |
| --- | --- | --- | --- |
| **TITLE** | | |  |
| Title | 1 | Identify the report as a systematic review. | Page 1 |
| **ABSTRACT** | | |  |
| Abstract | 2 | See the PRISMA 2020 for Abstracts checklist. | Page 3 |
| **INTRODUCTION** | | |  |
| Rationale | 3 | Describe the rationale for the review in the context of existing knowledge. | Page 5 |
| Objectives | 4 | Provide an explicit statement of the objective(s) or question(s) the review addresses. | Page 5 |
| **METHODS** | | |  |
| Eligibility criteria | 5 | Specify the inclusion and exclusion criteria for the review and how studies were grouped for the syntheses. | Page 6 |
| Information sources | 6 | Specify all databases, registers, websites, organisations, reference lists and other sources searched or consulted to identify studies. Specify the date when each source was last searched or consulted. | Pages 5 & 6 |
| Search strategy | 7 | Present the full search strategies for all databases, registers and websites, including any filters and limits used. | Page 5 & Table S1 |
| Selection process | 8 | Specify the methods used to decide whether a study met the inclusion criteria of the review, including how many reviewers screened each record and each report retrieved, whether they worked independently, and if applicable, details of automation tools used in the process. | Pages 6 & 7 |
| Data collection process | 9 | Specify the methods used to collect data from reports, including how many reviewers collected data from each report, whether they worked independently, any processes for obtaining or confirming data from study investigators, and if applicable, details of automation tools used in the process. | Pages 6 & 7 |
| Data items | 10a | List and define all outcomes for which data were sought. Specify whether all results that were compatible with each outcome domain in each study were sought (e.g. for all measures, time points, analyses), and if not, the methods used to decide which results to collect. | Pages 6,7 & 8 |
|  | 10b | List and define all other variables for which data were sought (e.g. participant and intervention characteristics, funding sources). Describe any assumptions made about any missing or unclear information. | Pages 6, 7 & 8, Table S3, Figures S1-S3 |
| Study risk of bias assessment | 11 | Specify the methods used to assess risk of bias in the included studies, including details of the tool(s) used, how many reviewers assessed each study and whether they worked independently, and if applicable, details of automation tools used in the process. | Page 8 |
| Effect measures | 12 | Specify for each outcome the effect measure(s) (e.g. risk ratio, mean difference) used in the synthesis or presentation of results. | Page 7 & 8 |
| Synthesis methods | 13a | Describe the processes used to decide which studies were eligible for each synthesis (e.g. tabulating the study intervention characteristics and comparing against the planned groups for each synthesis (item #5)). | Page 6 & 7 Table S3 |
|  | 13b | Describe any methods required to prepare the data for presentation or synthesis, such as handling of missing summary statistics, or data conversions. | Pages 6 & 7 |
|  | 13c | Describe any methods used to tabulate or visually display results of individual studies and syntheses. | Page 7 |
|  | 13d | Describe any methods used to synthesize results and provide a rationale for the choice(s). If meta-analysis was performed, describe the model(s), method(s) to identify the presence and extent of statistical heterogeneity, and software package(s) used. | Page 7 |
|  | 13e | Describe any methods used to explore possible causes of heterogeneity among study results (e.g. subgroup analysis, meta-regression). | Page 8 |
|  | 13f | Describe any sensitivity analyses conducted to assess robustness of the synthesized results. | N/A |
| Reporting bias assessment | 14 | Describe any methods used to assess risk of bias due to missing results in a synthesis (arising from reporting biases). | Page 8 |
| Certainty assessment | 15 | Describe any methods used to assess certainty (or confidence) in the body of evidence for an outcome. | Page 8 |
| **RESULTS** | | |  |
| Study selection | 16a | Describe the results of the search and selection process, from the number of records identified in the search to the number of studies included in the review, ideally using a flow diagram. | Pages 7 & 8 |
|  | 16b | Cite studies that might appear to meet the inclusion criteria, but which were excluded, and explain why they were excluded. | N/A |
| Study characteristics | 17 | Cite each included study and present its characteristics. | Pages 7 & 8, Table S3 |
| Risk of bias in studies | 18 | Present assessments of risk of bias for each included study. | Page 8, Figures 1, 2, S4 & S5 |
| Results of individual studies | 19 | For all outcomes, present, for each study: (a) summary statistics for each group (where appropriate) and (b) an effect estimate and its precision (e.g. confidence/credible interval), ideally using structured tables or plots. | Pages 8, 9, 10 and Figures 3-15, Figures S6, S7 |
| Results of syntheses | 20a | For each synthesis, briefly summarise the characteristics and risk of bias among contributing studies. | Pages 10-15 and Figures 3-15, Figures S6, S7 |
|  | 20b | Present results of all statistical syntheses conducted. If meta-analysis was done, present for each the summary estimate and its precision (e.g. confidence/credible interval) and measures of statistical heterogeneity. If comparing groups, describe the direction of the effect. | Pages 10-15 and Figures 3-15, Supplementary File 2 |
|  | 20c | Present results of all investigations of possible causes of heterogeneity among study results. | Figures 3-15, Supplementary File 2 |
|  | 20d | Present results of all sensitivity analyses conducted to assess the robustness of the synthesized results. | N/A |
| Reporting biases | 21 | Present assessments of risk of bias due to missing results (arising from reporting biases) for each synthesis assessed. | Figures 1 & 2, Pages 10-15 |
| Certainty of evidence | 22 | Present assessments of certainty (or confidence) in the body of evidence for each outcome assessed. | Pages 15 & 16, Supplementary File 3 |
| **DISCUSSION** | | |  |
| Discussion | 23a | Provide a general interpretation of the results in the context of other evidence. | Pages 16 & 17 |
|  | 23b | Discuss any limitations of the evidence included in the review. | Page 23 & 24 |
|  | 23c | Discuss any limitations of the review processes used. | Page 23 & 24 |
|  | 23d | Discuss implications of the results for practice, policy, and future research. | Pages 19 |
| **OTHER INFORMATION** | | |  |
| Registration and protocol | 24a | Provide registration information for the review, including register name and registration number, or state that the review was not registered. | Page 5 |
|  | 24b | Indicate where the review protocol can be accessed, or state that a protocol was not prepared. | Page 5 |
|  | 24c | Describe and explain any amendments to information provided at registration or in the protocol. | Page 5 |
| Support | 25 | Describe sources of financial or non-financial support for the review, and the role of the funders or sponsors in the review. | Page 28 |
| Competing interests | 26 | Declare any competing interests of review authors. | Page 28 |
| Availability of data, code and other materials | 27 | Report which of the following are publicly available and where they can be found: template data collection forms; data extracted from included studies; data used for all analyses; analytic code; any other materials used in the review. | Page 28 |

# Table S3. Eligible study characteristics

| # | Author | Year | N | Specimen | Biomarker | Exercise protocol | PMID |
| --- | --- | --- | --- | --- | --- | --- | --- |
| 1 | Allgrove et al. | 2011 | 20 | Plasma/serum | F2-isoprostanes | Cycling at 60% VO2max for 90 min, with the intensity increased to 90% VO2max for a 30-s period every 10-min, followed by a ride to exhaustion at 90% VO2max. | 21558573 |
| 2 | Bailey et al. | 2011 | 18 | Urine | F2-isoprostanes | 90-min of intermittent shuttle running at VO2max equally divided into 6 blocks. | 21069377 |
| 3 | Barbaresi et al. | 2021 | 14 | Erythrocyte | Glutathione | VO2max cycling test, starting at 100 W for 2-min and with an incremental rate of 50 W/2-min, the subjects maintained a constant pedal cadence of 80 rpm until volitional exhaustion. | 33588872 |
| 4 | Berzosa et al. | 2011 | 34 | Plasma/serum | Protein carbonyls | 3 cycloergometric tests: a continuous progressive exercise, a strenuous exercise until exhaustion and an acute bout of exercise at an intensity corresponding to 70% of maximal work capacity for 30-min. | 21116825 |
| 5 | Bloomer et al. | 2009 | 14 | Plasma/serum | Protein carbonyls | Walking (speed: 3-3.5 mph and grade: 0-15%, both gradually increased) on a treadmill while carrying 25% of body mass for 60-min. | 19691834 |
| 6 | Broome et al. | 2021 | 16 | Plasma/serum | F2-isoprostanes | Cycle ergometer, 10 × 60-s at VO2peak workload with 75-s rest. | 34419082 |
| 7 | Ceci et al. | 2015 | 13 | Plasma/serum | Protein carbonyls | Cycling ergometer with pedaling rate at 60 r/min, and then the workload was increased by 30 watt every 3-min until exhaustion. | 25381629 |
| 8 | Chaouachi et al. | 2022 | 8 | Plasma/serum | F2-isoprostanes | Yoyo Intermittent Recovery Test Level 2. | 35394687 |
| 9 | Chung et al. | 2023 | 35 | Erythrocyte | Glutathione | 30-min running at 60% VO2max. | 36727618 |
| 10 | De Marchi et al. | 2012 | 22 | Plasma/serum | Protein carbonyls | Treadmill running, starting with 3 km/h for 3 min and afterwards the velocity was increased 1 km/h every minute until 16 km/h and performed until exhaustion. | 21739259 |
| 11 | De Marchi et al. | 2017 | 8 | Plasma/serum | Protein carbonyls | 5 sets of 10 eccentric/concentric contractions of the elbow flexors separated by 30-s. Contractions were performed with an amplitude of 90° and speed of 90°/seg for the eccentric contractions and 180°/seg for concentric contractions. | 28054262 |
| 12 | Deli et al. | 2018 | 11 | Erythrocyte | Glutathione | 90-min constant-intensity (60–70% VO2max) submaximal cycling. | 29469654 |
| 13 | Deminice et al. | 2013 | 12 | Erythrocyte | Glutathione | 2 consecutive Running-based Anaerobic Sprint Tests (RAST). RAST consisted of six 35-m sprint runs at maximum speed with 10-s rest between. | 23800565 |
| 14 | Diaz-Castro et al. | 2012 | 20 | Urine | F2-isoprostanes | Combination of mountain run and ultra-endurance, in a constant run with a total distance of 50 km, from an initial altitude of 640 m above sea level to an altitude of 3,393 m. | 22212862 |
| 15 | Diaz-Castro et al. | 2012 | 10 | Urine | F2-isoprostanes | Combination of mountain run and ultra-endurance, in a constant run with a total distance of 50 km, from an initial altitude of 640 m above sea level to an altitude of 3,393 m. | 21990004 |
| 16 | Djordjevic et al. | 2012 | 14 | Erythrocyte | Glutathione | Maximal progressive exercise on a cycle ergometer. Athletes were instructed to keep the revolution rate at 60 rpm. The load was set to 2 W/kg and increased by 50 W every 3-min until voluntary exhaustion. | 23304255 |
| 17 | Draganidis et al. | 2017 | 11 | Erythrocyte | Protein carbonyls | 300 eccentric unilateral contractions (20 sets, 15 repetitions/set, 30-s rest between sets) of knee extensors on an isokinetic dynamometer at a speed of 30°/s. | 28831951 |
| 18 | Fatouros et al. | 2010 | 10 | Erythrocyte, Plasma/serum | Glutathione, protein carbonyls | Football match | 19996787 |
| 19 | Filip-Stachnik et al. | 2023 | 10 | Erythrocyte | Glutathione | a) leg press, b) sitting leg extension, c) lying leg curls, d) lat pull-down, e) chest supported row, f) Smith machine bench press, and g) overhead machine press. In each exercise, participants performed three sets at 70% of their 1RM until volitional failure. | 37711718 |
| 20 | Flanagan et al. | 2018 | 10 | Erythrocyte | Glutathione | Leg press for 5 sets of 12 repetitions at 70% of 1RM, with 2-min rest between sets. | 28981384 |
| 21 | Gholami et al. | 2021 | 11 | Plasma/serum | F2-isoprostanes | 3-min warm-up running at 3 km/h, with speed increased to 6 km/h for 3-min and then the speed increased 1 km/h every minute until failure. | 33639967 |
| 22 | Goldfarb et al. | 2011 | 20 | Plasma/serum | Protein carbonyls | 4 sets of 12 repetitions using their nondominant arm elbow flexors, at an angular velocity of 20°/s. The range of motion was set from full extension to 100° of flexion. | 20689455 |
| 23 | Goldfarb et al. | 2005 | 9 | Plasma/serum | Protein carbonyls | 4 sets of 12 repetitions using their nondominant arm elbow flexors, at an angular velocity of 20°/s. The range of motion was set from full extension to 100° of flexion. | 15692318 |
| 24 | Goldfarb et al. | 2005 | 12 | Plasma/serum | Protein carbonyls | Graded maximal oxygen consumption test on a motorized treadmill. 5-min warm-up and the workload was increased every 2-min and the test was stopped when the VO2 or HR leveled off or the subject was fatigued. | 16131698 |
| 25 | Gray et al. | 2014 | 10 | Plasma/serum | Protein carbonyls | 20 sets of 10 eccentric contractions at 0.52 rads/s between 90° and 120°. Each set was separated by 120-s. | 24225668 |
| 26 | Howatson et al. | 2010 | 10 | Plasma/serum | Protein carbonyls | Marathon running. | 19883392 |
| 27 | Hudson et al. | 2008 | 10 | Plasma/serum | Protein carbonyls | 2 different back squat protocols: 1) a hypertrophy protocol of 4 sets, 10 repetitions with 90-s of rest at 75% 1RM and 2) a strength protocol of 11 sets, 3 repetitions with 5-min of rest at 90% 1RM. | 18379219 |
| 28 | Jamurtas et al. | 2018 | 12 | Plasma/serum | Protein carbonyls | The HIIT exercise bout involved the performance of four 30-sec sprints on a cycle ergometer (against a resistance of 0.375 kg/kg of body mass) interspersed with 4 min of recovery. | 30116124 |
| 29 | Jamurtas et al. | 2006 | 9 | Erythrocyte | Glutathione | 45-min on the treadmill at 70-75% of HRmax. | 17203782 |
| 30 | Kaikkonen et al. | 1998 | 19 | Erythrocyte | Glutathione | Marathon running. | 9733025 |
| 31 | Kalafati et al. | 2010 | 9 | Erythrocyte, Plasma/serum | Glutathione, protein carbonyls | Treadmill running at 70%–75% of VO2max for 2h. After the 2h run, the speed of the treadmill was increased to 95% VO2max and exercise was terminated at exhaustion. | 20010119 |
| 32 | Kerasioti et al | 2012 | 9 | Erythrocyte, Plasma/serum | Glutathione, protein carbonyls | 2-h exercise on a cycle ergometer at 60–65% VO2max. | 22538083 |
| 33 | Knab et al. | 2013 | 9 | Plasma/serum | Protein carbonyls, F2-isoprostanes | Workout consisted of a 30 to 45-min warm-up followed by 45-min of high-intensity interval training and 15 to 25-min of cool-down swimming (leg kicks and technique drills). Total practice time was 2-h and consisted of approximately 5500-m swim interval training. | 23070789 |
| 34 | Kouvelioti et al. | 2019 | 20 | Plasma/serum | Protein carbonyls | Twenty healthy men (22.3 ± 2.3 years) performed two high-intensity interval exercise trials (crossover design); running on treadmill and cycling on cycle ergometer. Trials consisted of eight 1 min running or cycling intervals at ≥ 90% of maximal heart rate, separated by 1 min passive recovery intervals | 30671591 |
| 35 | Kritikos et al. | 2021 | 10 | Erythrocyte, Plasma/serum | Glutathione, protein carbonyls | Speed-endurance training (60-min) was performed on natural grass and incorporated 1 set of 8 (30-s each) maximum-intensity repetitions with a passive recovery of 2.5-min. | 33726784 |
| 36 | Kyparos et al. | 2012 | 9 | Erythrocyte, Plasma/serum | Glutathione, protein carbonyls | 2000-m all out rowing | 21947454 |
| 37 | Kyparos et al. | 2009 | 19 | Erythrocyte, Plasma/serum | Glutathione, protein carbonyls | 2000-m rowing ergometer race | 19620924 |
| 38 | Kyparos et al. | 2007 | 11 | Erythrocyte, Plasma/serum | Glutathione, protein carbonyls | Volitional fatigue test of shuttle runs within the tennis court hitting the ball until exhaustion | 18091691 |
| 39 | Leeder et al. | 2014 | 8 | Plasma/serum | F2-isoprostanes | 5 sets of approximately 15-min of varying-intensity exercise ranging from walking to jogging (55% VO2max), running (95% VO2max) and sprinting. At the end of the test, participants completed a progressive shuttle-run test to exhaustion. | 23479406 |
| 40 | Margaritelis et al. | 2018 | 12 | Erythrocyte, Plasma/serum, Urine | Glutathione, protein carbonyls, F2-isoprostanes | After a 5-min warm-up period at 100 W on the cycle ergometer, workload increased by 50 W every 2.5-min until heart rate reached 160 bpm. Then, workload increased by 25 W every 2.5-min until exhaustion. The test was terminated when 3 of the following 4 criteria were met: (i) volitional fatigue, (ii) a lower than 2 mL/kg/min increase in VO2 despite an increase in workload, (iii) a respiratory exchange ratio greater than or equal to 1.10 and (iv) heart rate within 10 bpm of the predicted maximal heart rate (220–age). | 28544643 |
| 41 | Margaritelis et al. | 2016 | 12 | Urine | F2-isoprostanes | After a 5-min warm-up period at 100 W on the cycle ergometer, workload increased by 50 W every 2.5-min until heart rate reached 160 bpm. Then, workload increased by 25 W every 2.5-min until exhaustion. The test was terminated when 3 of the following 4 criteria were met: (i) volitional fatigue, (ii) a lower than 2 mL/kg/min increase in VO2 despite an increase in workload, (iii) a respiratory exchange ratio greater than or equal to 1.10 and (iv) heart rate within 10 bpm of the predicted maximal heart rate (220–age). | 27596985 |
| 42 | Margaritelis et al. | 2014 | 98 | Erythrocyte, Plasma/serum, Urine | Glutathione, protein carbonyls, F2-isoprostanes | 5 sets of 8 eccentric maximal voluntary contractions [knee range, 0° (full extension) to 90° flexion] at an angular velocity of 60°/s on an isokinetic dynamometer. A 2-min rest interval was used between sets. | 24634834 |
| 43 | Martinez-Noguera et al. | 2019 | 15 | Erythrocyte | Glutathione | Repeated sprint test (4x 30'') with 5-min rest. | 31416212 |
| 44 | Mastaloudis et al. | 2004 | 10 | Plasma/serum | F2-isoprostanes | Treadmill running at a moderate pace; every 3-min the grade and intensity were increased until subjects achieved 85% of their age-predicted HRmax | 15110397 |
| 45 | McAllister et al. | 2018 | 14 | Erythrocyte | Glutathione | 35-min of steady-state cycling at 60% VO2 peak. | 30580652 |
| 46 | McAnulty et al. | 2007a | 15 | Plasma/serum | F2-isoprostanes | Treadmill running for 45-min at 80% of VO2max. | 18059589 |
| 47 | McAnulty et al. | 2003 | 16 | Plasma/serum | F2-isoprostanes | Treadmill running for 3-h at 70% of VO2max. | 14567443 |
| 48 | McAnulty et al. | 2010 | 12 | Plasma/serum | F2-isoprostanes | Cycling for 3-h at 57% Wmax. | 20164809 |
| 49 | McAnulty et al. | 2007b | 25 | Plasma/serum | F2-isoprostanes | 100-Mile Endurance Run. | 17596774 |
| 50 | McAnulty et al. | 2007c | 15 | Plasma/serum | F2-isoprostanes | Cycling for 3-h at 60% Wmax and 75% VO2peak. | 17497585 |
| 51 | McAnulty et al. | 2005a | 6 | Plasma/serum | F2-isoprostanes | Low-intensity exercise (50% VO2max) on a treadmill until a core temperature of 39.5 °C was reached. | 15776334 |
| 52 | McAnulty et al. | 2007d | 12 | Plasma/serum | F2-isoprostanes | Cycling for 2-h at 60-65% Wmax or 75% VO2peak. | 18046057 |
| 53 | McAnulty et al. | 2005b | 15 | Plasma/serum | F2-isoprostanes | The session consisted of 10 different exercises: flat bench press, incline bench press, military press, upright row, bent-over row, French curl, biceps curl, back squat, front squat and deadlift. Subjects performed 4 sets of 10 repetitions for each resistance exercise, with the first set at 40% of the subject’s 1-RM and the subsequent sets at 60% 1-RM. Two-minute rest intervals separated sets for the bench press, incline bench press, military press, upright row, bent-over row, French curl and biceps curl. Three-minute rest intervals were given following the completion of each exercise and between sets for the back squat, front squat and deadlift. | 16298748 |
| 54 | McAnulty et al. | 2008 | 17 | Plasma/serum | F2-isoprostanes | Cycling for 3-h at 57% Wmax. | 18347680 |
| 55 | McAnulty et al. | 2013 | 11 | Plasma/serum | Protein carbonyls | 1-h run at a 3% grade and 80% VO2max. | 23980734 |
| 56 | McGinnis et al. | 2014 | 11 | Plasma/serum | Protein carbonyls | A 60-min exercise trial with intensity prescribed at 60% VO2peak measured at 975 m and was performed in a normoxic environment at 975 m | 24667140 |
| 57 | Medved et al. | 2004 | 8 | Skeletal muscle | Glutathione | Cycling at 70% VO2 peak for 45-min and then to volitional fatigue at 90% VO2 peak. | 15194675 |
| 58 | Merry et al. | 2010 | 9 | Skeletal muscle | Glutathione | 80-min of cycling at 62±1% of VO2peak | 20308250 |
| 59 | Michailidis et al. | 2013 | 10 | Skeletal muscle | Glutathione, protein carbonyls | 300 eccentric unilateral repetitions (20 sets, 15 repetitions/set, 30-s rest between sets) with the quadriceps muscle group at a speed of 30°/s on an isokinetic dynamometer | 23719546 |
| 60 | Michailidis et al. | 2007 | 11 | Erythrocyte, Plasma/serum | Glutathione, protein carbonyls | 45 min at 70–75% VO2max and then at 90% VO2max to exhaustion on a treadmill | 17596778 |
| 61 | Morillas-Ruiz et al. | 2005 | 13 | Plasma/serum | Protein carbonyls | 90-min constant-load test on a bicycle ergometer at 70% VO2 max. | 16132121 |
| 62 | Morrison et al. | 2015 | 5 | Skeletal muscle | Glutathione, F2-isoprostanes | 10 x 4-min cycling intervals at 90% of VO2peak, interspersed with 2-min of active rest at 50 W. | 26482865 |
| 63 | Mullins et al. | 2013 | 30 | Plasma/serum | Protein carbonyls, F2-isoprostanes | The Bruce treadmill protocol | 23862764 |
| 64 | Nakhostin-Roohi et al. | 2011 | 9 | Plasma/serum | Protein carbonyls | 14-km running | 21899544 |
| 65 | Nieman et al. | 2013 | 15 | Plasma/serum | Protein carbonyls | Treadmill running for 2.5-h at approximately 70% VO2max | 23967286 |
| 66 | Nieman et al. | 2016 | 10 | Plasma/serum | F2-isoprostanes | Treadmill running at 70% of VO2max for 1.5-h followed by 30-min of downhill running on a 10% grade the same intensity. | 27018002 |
| 67 | Nieman et al. | 2014a | 19 | Plasma/serum | F2-isoprostanes | 75-km cycling | 24760997 |
| 68 | Nieman et al. | 2004 | 17 | Plasma/serum | F2-isoprostanes | Triathlon (3.9-km ocean swim, 180-km bike race and 42-km run). | 15292740 |
| 69 | Nieman et al. | 2014b | 18 | Plasma/serum | F2-isoprostanes | 75-km cycling | 25409020 |
| 70 | Nikolaidis et al | 2006 | 9 | Erythrocyte, Plasma/serum | Glutathione, protein carbonyls | 45 min at 70–75% VO2max and then at 90% VO2max to exhaustion on a treadmill | 16888458 |
| 71 | Nikolaidis et al. | 2012 | 10 | Erythrocyte, Plasma/serum, Urine | Glutathione, protein carbonyls, F2-isoprostanes | 45-min of running at an intensity corresponding to 70%–75% of VO2max. | 22288504 |
| 72 | Nikolaidis et al. | 2013 | 10 | Erythrocyte, Plasma/serum, Urine | Glutathione, protein carbonyls, F2-isoprostanes | 5 sets of 8 eccentric maximal voluntary contractions (MVC) [knee range, 0° (full extension) to 90° flexion] at an angular velocity of 60°/s. A 2-min rest interval was utilized between sets. | 23628501 |
| 73 | Palazzetti et al. | 2004 | 10 | Erythrocyte | Glutathione | Duathlon test (run 5 km, cycle 20 km, run 5 km). | 14748941 |
| 74 | Panza et al. | 2016 | 12 | Erythrocyte | Glutathione | 3 sets of 20 repetitions of unilateral maximal isokinetic eccentric actions of the elbow flexor muscles at an angular velocity of 45°/s, with 2-min rest between sets. | 26917157 |
| 75 | Panza et al. | 2008 | 14 | Erythrocyte | Glutathione | Bench press exercise for 4 sets of 10, 8, 6, and 4 repetitions, with 75%, 80%, 85% and 90% of 1RM, respectively with 1.5-min intervals between repetitions. | 18337059 |
| 76 | Papapanagiotou et al. | 2011 | 18 | Plasma/serum | F2-isoprostanes | Hockey match | 21480058 |
| 77 | Pappas et al. | 2021 | 12 | Plasma/serum | Protein carbonyls | 5 sets of 15 eccentric maximal voluntary contractions (knee range, 0° full extension to 90° flexion) at an angular velocity of 60°/s. A 2-min rest interval was used between sets and the total workout time was 15-min. | 33808079 |
| 78 | Paschalis et al. | 2016 | 10 | Erythrocyte, Plasma/serum, Urine | Glutathione, protein carbonyls, F2-isoprostanes | Cycling in ergometer at an intensity corresponding to 70–75 % of the subject’s VO2max for 45-min at 50 rpm. Following the 45-min cycling, the intensity of the cycle ergometer was increased to elicit 95 % VO2max, and exercise was terminated at exhaustion | 25526969 |
| 79 | Paschalis et al. | 2007 | 10 | Erythrocyte, Plasma/serum | Glutathione, protein carbonyls | 5 sets of 15 eccentric maximal voluntary leg extensions in the seated position with 1-min rest interval between sets. | 18019428 |
| 80 | Pinto et al. | 2022 | 12 | Plasma/serum | Protein carbonyls | 21-15-9 Crossfit WOD with 2 exercises: hang squat clean and box jump over. | 35910832 |
| 81 | Poulios et al. | 2018 | 20 | Erythrocyte | Glutathione, protein carbonyls | Football match. | 29659539 |
| 82 | Quindry et al. | 2008 | 22 | Plasma/serum | Protein carbonyls, F2-isoprostanes | 160-km endurance run. | 19164830 |
| 83 | Quindry et al. | 2013 | 12 | Plasma/serum | Protein carbonyls | 1-h cycling at 60% Wmax. | 23532145 |
| 84 | Quinn et al. | 2021 | 14 | Plasma/serum | F2-isoprostanes | Treadmill running with a speed leading to intensity that elicited 80% of HRmax for 25-min. | 33172351 |
| 85 | Rietjens et al. | 2007 | 7 | Urine | F2-isoprostanes | 8 sets of 10 repetitions on the horizontal leg press machine and 8 sets of 10 repetitions on the leg extension machine at 75% of the individual 1RM, with 2-min rest intervals between sets. | 18046185 |
| 86 | Rosvoglou et al. | 2023 | 10 | Erythrocyte, Plasma/serum | Glutathione, protein carbonyls | 300 (20 sets, 15 reps) maximal eccentric unilateral repetitions with the quadriceps muscle at a speed of 60°/s on an isokinetic dynamometer with a 30-s rest between sets. | 37864292 |
| 87 | Sacheck et al. | 2003 | 8 | Plasma/serum | F2-isoprostanes | Downhill running on a treadmill for 3 successive 15-min intervals (with a 5-min rest between bouts) at a speed that corresponded to 75% of VO2max. | 12788477 |
| 88 | Serravite et al. | 2014 | 8 | Plasma/serum | Protein carbonyls | 10 sets of 10 lengthening contractions on a seated preacher-curl bench using a dumbbell loaded at 120% 1RM. The dumbbell was lowered from 75° to 180° at 5 s/repetition tempo. | 24662009 |
| 89 | Silva et al. | 2014 | 10 | Plasma/serum | Protein carbonyls | Eccentric exercise was performed with elbow flexion and extension on the Scott bench at an intensity of 80% of 1RM. The concentric phase (flexion) of the exercise was performed with manual assistance from the instructor. The eccentric phase (extension) was performed for a duration of 6 to 8-s. 3 sets of exercises were performed with 2-min rest intervals. | 24383513 |
| 90 | Silva et al. | 2010 | 10 | Plasma/serum | Protein carbonyls | Eccentric exercise was performed with elbow flexion and extension on the Scott bench at an intensity of 80% of 1RM. The concentric phase (flexion) of the exercise was performed with manual assistance from the instructor. The eccentric phase (extension) was performed for a duration of 6 to 8-s. 3 sets of exercises were performed with 2-min rest intervals. | 19859781 |
| 91 | Silva et al. | 2008 | 8 | Plasma/serum | Protein carbonyls | Eccentric exercise was performed with elbow flexion and extension on the Scott bench at an intensity of 80% of 1RM. The concentric phase (flexion) of the exercise was performed with manual assistance from the instructor. The eccentric phase (extension) was performed for a duration of 6 to 8-s. 3 sets of exercises were performed with 2-min rest intervals. | 18708687 |
| 92 | Skarpañska-Stejnborn et al. | 2008 | 10 | Erythrocyte | Glutathione | 2000 m rowing exercise test. | 18562776 |
| 93 | Steensberg et al. | 2002 | 11 | Plasma/serum | F2-isoprostanes | Treadmill running for 2.5-h at 75% of VO2max. | 12012074 |
| 94 | Sureda et al. | 2013 | 7 | Neutrophils | Protein carbonyls | Half-marathon running. | 23400620 |
| 95 | Theodorou et al. | 2011 | 14 | Erythrocyte, Plasma/serum | Glutathione, protein carbonyls | 5 sets of 15 eccentric maximal voluntary contractions with each leg at an angular velocity of 60/s in the seated position. A 2-min rest interval was incorporated between sets. | 21508092 |
| 96 | Theodorou et al. | 2010 | 9 | Erythrocyte, Plasma/serum | Glutathione, protein carbonyls | 5 sets of 15 eccentric maximal voluntary contractions with each leg at an angular velocity of 60/s in the seated position. A 2-min rest interval was incorporated between sets. | 19997026 |
| 97 | Tomazoni et al. | 2019 | 22 | Plasma/serum | Protein carbonyls | Treadmill running with speed of 3km/h during the first 3-min. After this, speed was increased 1 km/h every minute until 16 km/h and exercise was performed until exhaustion. | 31827687 |
| 98 | Trewin et al. | 2015 | 7 | Skeletal muscle | Glutathione, protein carbonyls | 55-min bout of cycling exercise at 65% of VO2peak. Following this, the workload was increased to 85% of VO2peak for the final 5-min. | 26105008 |
| 99 | Trewin et al. | 2013 | 9 | Erythrocyte | Glutathione | 6 x 5-min high-intensity intermitent cycling bouts separated by 1-min of active recovery at 100 W. | 24195622 |
| 100 | Wadley et al. | 2017 | 12 | Plasma/serum | Protein carbonyls | Standardized 10-min warm-up at 55% relative VO2max, followed by 75-min at 70% relative VO2max on an electromagnetically braked cycle ergometer. | 27710149 |
| 101 | White et al. | 2013 | 10 | Plasma/serum | F2-isoprostanes | 10 x 3-min running intervals at 85% VO2peak on a motorised treadmill, each separated with 90-s recovery. | 23079864 |
| 102 | Zembron-Lacny et al. | 2010 | 15 | Erythrocyte, Plasma/serum | Glutathione, Protein carbonyls | Cycling until volitional exhaustion, eliciting VO2max. The test was incremental and progressive, all subjects commenced at a 50 W workload and it was increased by 50 W every 3-min until exhaustion at a constant pedal speed of 60 rpm. | 20354834 |
| 103 | Zembron-Lacny et al. | 2022 | 10 | Erythrocyte, Plasma/serum | Glutathione | VO2max test on a treadmill. | 35745133 |

# Table S4. Publication bias assessment

**Glutathione**

| **Overall** | **Subgroup** | **Egger’s test** |
| --- | --- | --- |
| Immediately post-exercise | | |
| 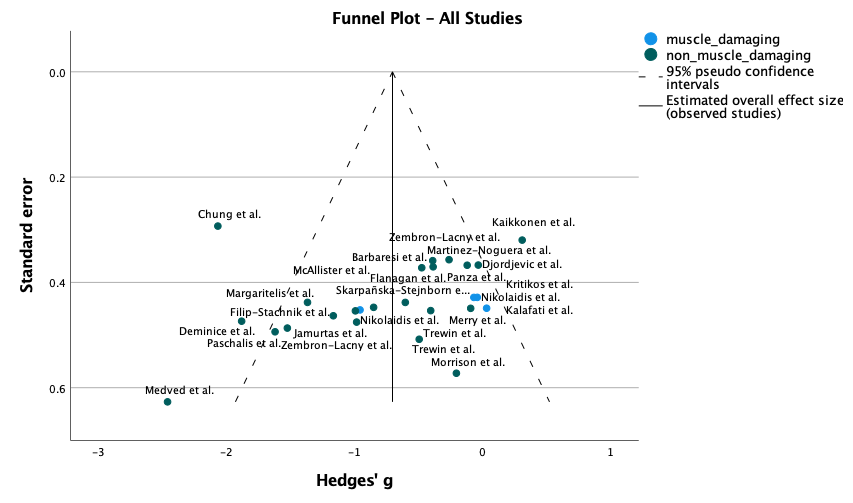 | 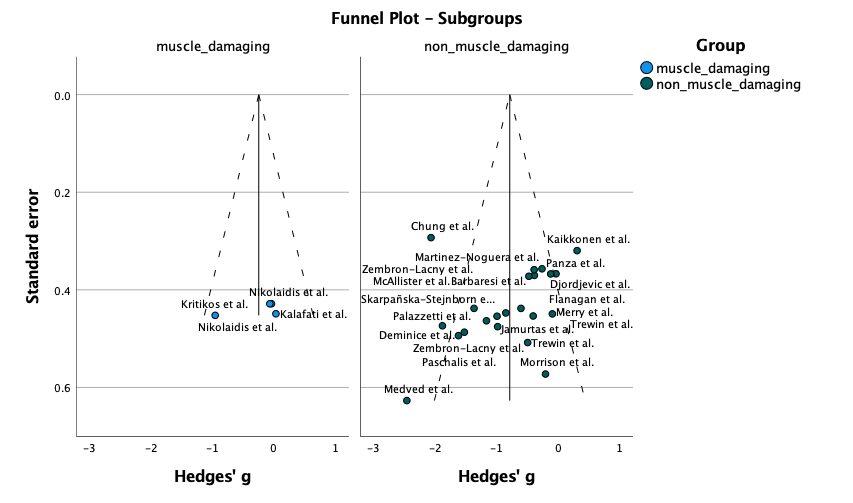 | Overall (k=32)  t=.830, p=.415  95%CI= [-1.027 – 2.407]  Non-muscle-damaging (k=27)  t=.792, p=.438  95%CI= [-1.090 – 2.424]  Muscle-damaging (k=5)  Non-applicable |
| 30min-2h post-exercise | | |
| 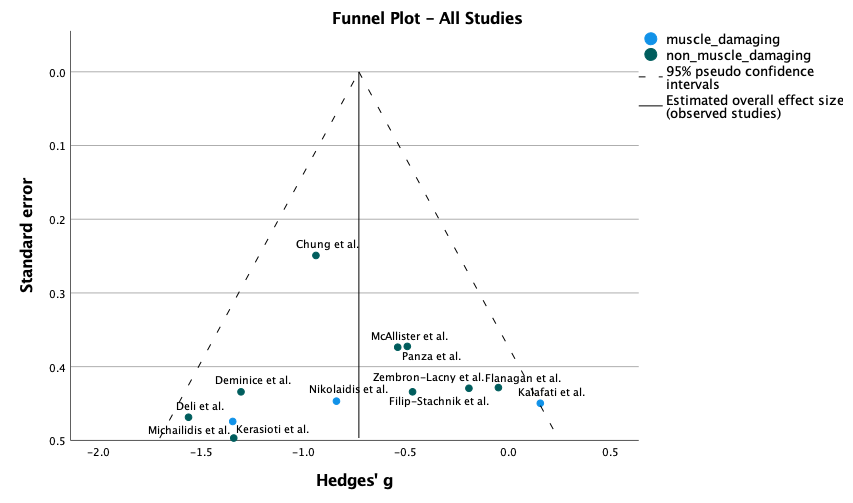 | 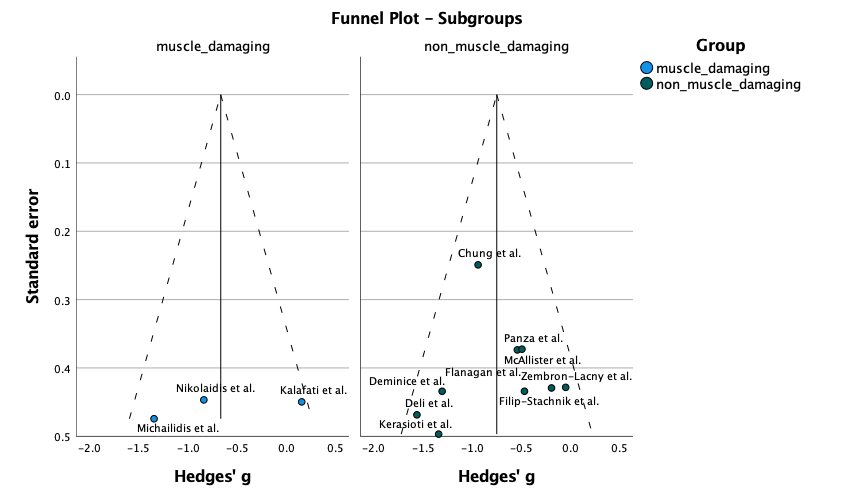 | Overall (k=13)  t=-.460, p=.656  95%CI= [-2.589 – 1.704]  Non-muscle-damaging (k=10)  t=-.485, p=.643  95%CI= [-2.769 – 1.827]  Muscle-damaging (k=3)  Non-applicable |
| 24h post-exercise | | |
| 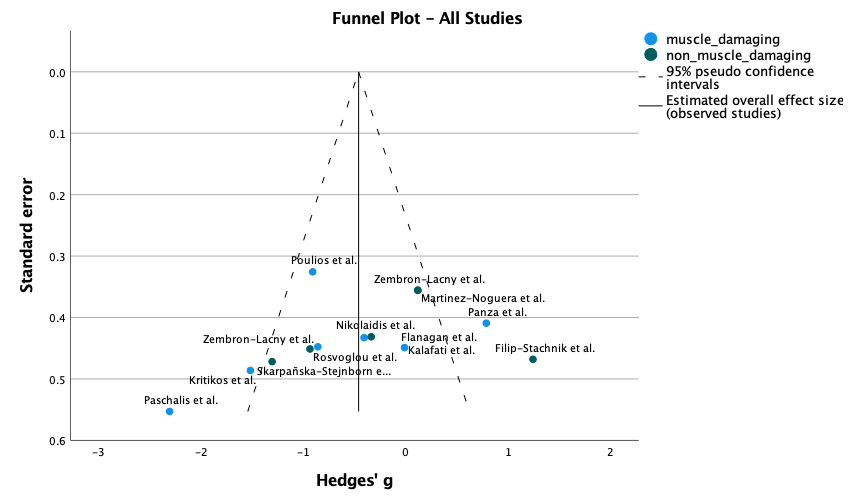 | Fewer than 10 studies were available for each exercise type (k=9 for muscle-damaging exercise; k=7 for non-muscle-damaging) | Overall (k=16)  t=1.335, p=.209  95%CI= [-1.571 – 6.413]  Non-muscle-damaging (k=7)  Non-applicable  Muscle-damaging (k=9)  Non-applicable |
| 48h post-exercise | | |
| 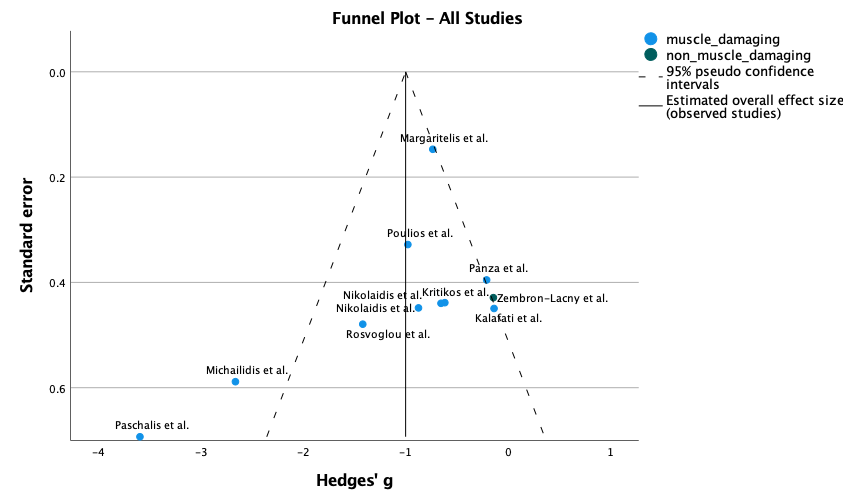 | 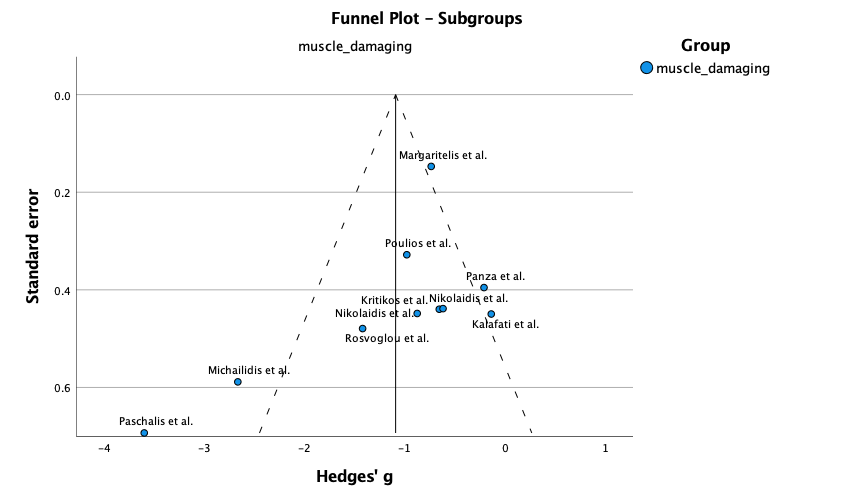 | Overall (k=13)  t=-.981, p=.352  95%CI= [-1.066 – 2.698]  Non-muscle-damaging (k=1)  Non-applicable  Muscle-damaging (k=12)  t=.895, p=.397  95%CI= [-1.154 – 2.618] |

**F_2_-isoprostanes**

| **Overall** | **Subgroup** | **Egger’s test** |
| --- | --- | --- |
| Immediately post-exercise | | |
| 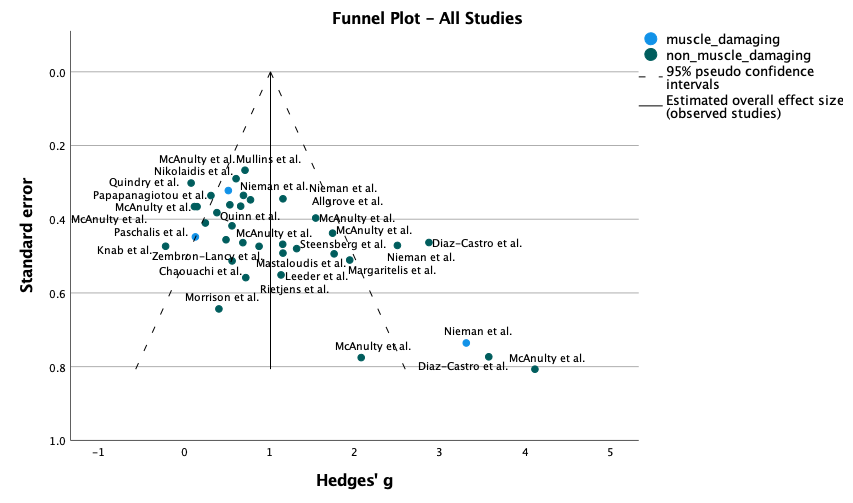 | 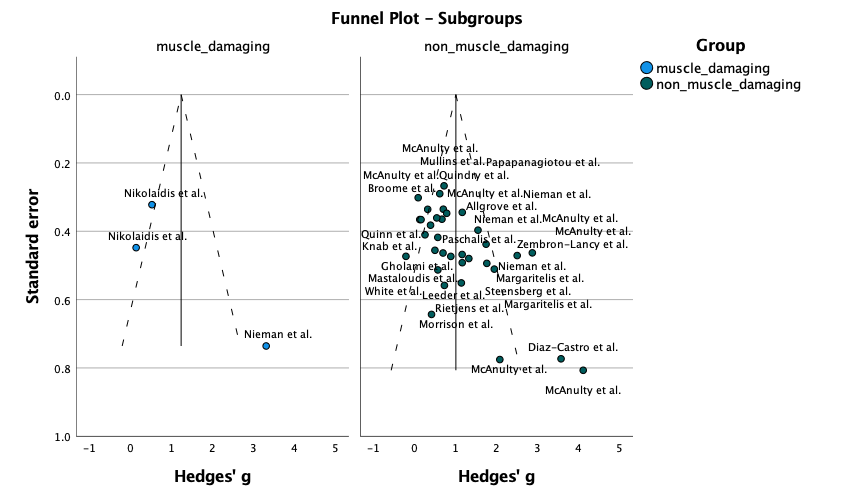 | Overall (k=37)  t=-2.502, **p=.017**  95%CI= [-2.005 – -.209]  Non-muscle-damaging (k=34)  t=-2.045, **p=.049**  95%CI= [-1.942 – -.004]  Muscle-damaging (k=3)  Non-applicable |
| 30min-2h post-exercise | | |
| 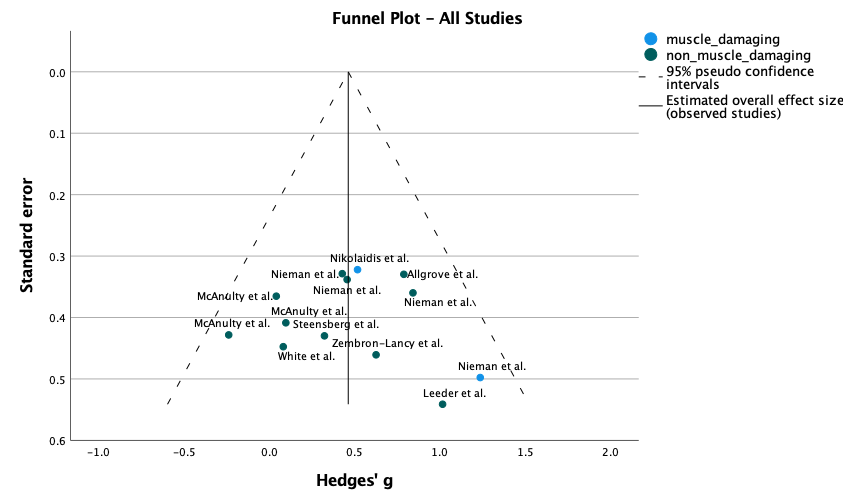 | 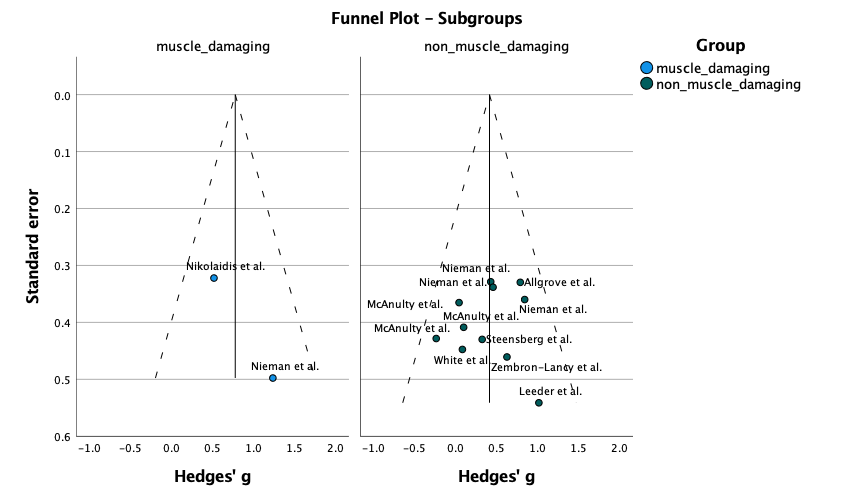 | Overall (k=13)  t=.425, p=.679  95%CI= [-1.222 – 1.806]  Non-muscle-damaging (k=11)  t=-.882, p=.401  95%CI= [-1.077 – 2.455]  Muscle-damaging (k=2)  Non-applicable |
| 24h post-exercise | | |
| Fewer than 10 studies were available for this time point in total (k=8) | Fewer than 10 studies were available for each exercise type (k=4 for muscle-damaging exercise; k=4 for non-muscle-damaging) | Overall (k=8)  Non-applicable  Non-muscle-damaging (k=4)  Non-applicable  Muscle-damaging (k=4)  Non-applicable |
| 48h post-exercise | | |
| Fewer than 10 studies were available for this time point in total (k=6) | Fewer than 10 studies were available for each exercise type (k=3 for muscle-damaging exercise; k=3 for non-muscle-damaging) | Overall (k=6)  Non-applicable  Non-muscle-damaging (k=3)  Non-applicable  Muscle-damaging (k=3)  Non-applicable |

**Protein carbonyls**

| **Overall** | **Subgroup** | **Egger’s test** |
| --- | --- | --- |
| Immediately post-exercise | | |
| 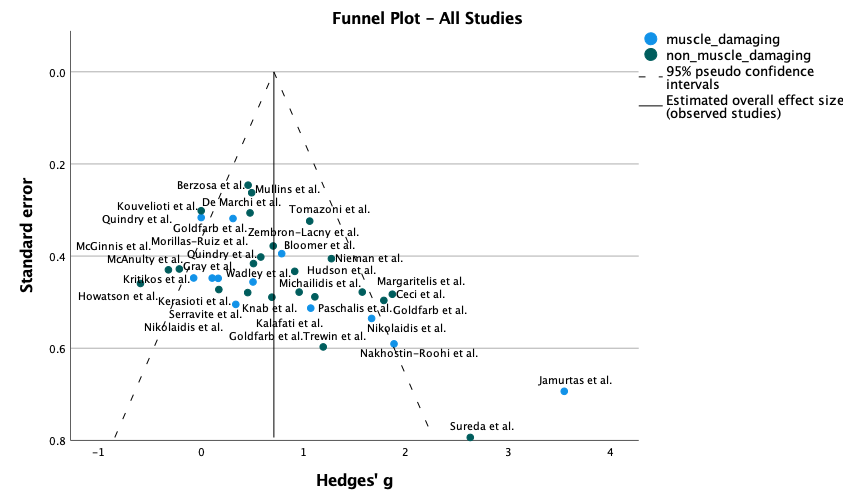 | 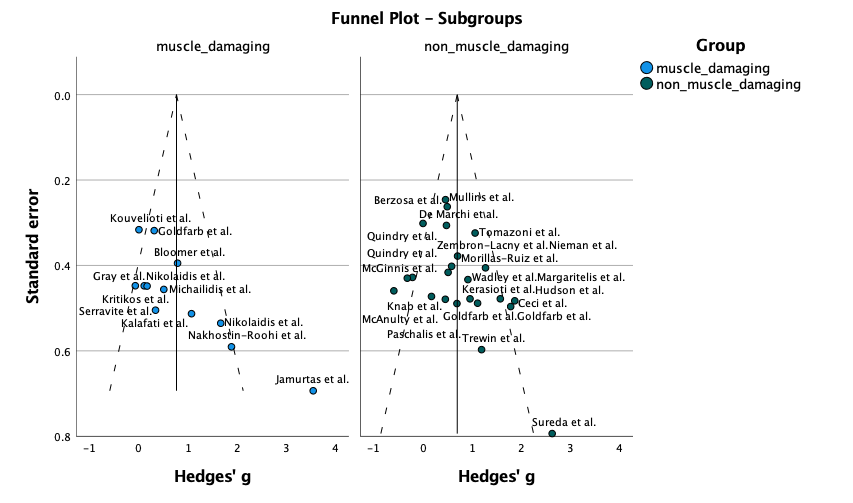 | Overall (k=40)  t=-2.248, **p=.031**  95%CI= [-1.930 – -.096]  Non-muscle-damaging (k=27)  t=-.936, p=.360  95%CI= [-1.607 – .610]  Muscle-damaging (k=13)  t=-3.019, **p=.013**  95%CI= [-4.110 – .620] |
| 30min-2h post-exercise | | |
| 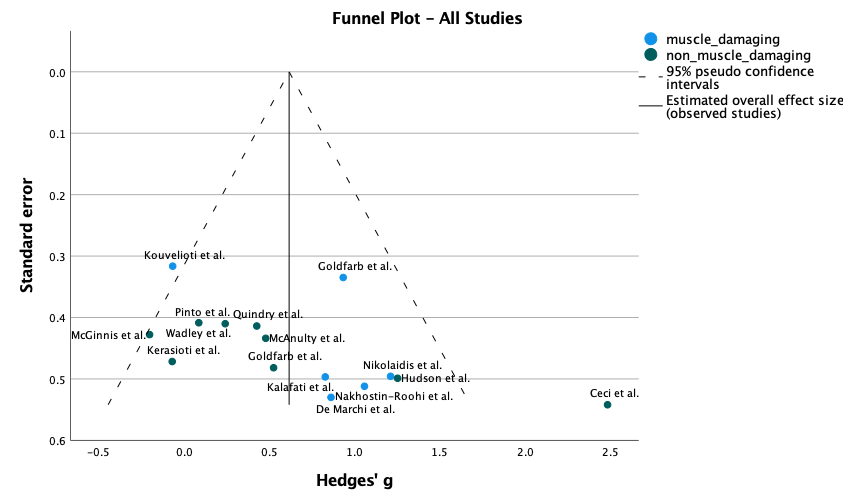 | Fewer than 10 studies were available for each exercise type (k=6 for muscle-damaging exercise; k=9 for non-muscle-damaging) | Overall (k=15)  t=-1.760, p=.102  95%CI= [-3.753 – .383]  Non-muscle-damaging (k=9)  Non-applicable  Muscle-damaging (k=6)  Non-applicable |
| 24h post-exercise | | |
| 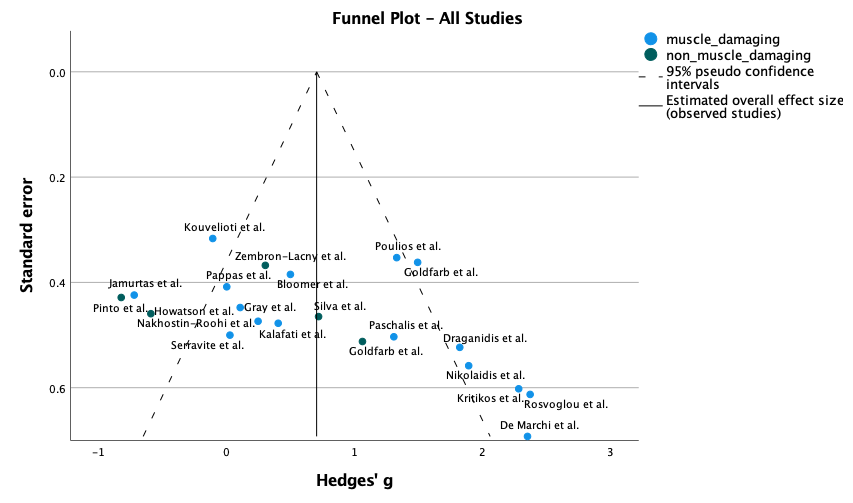 | 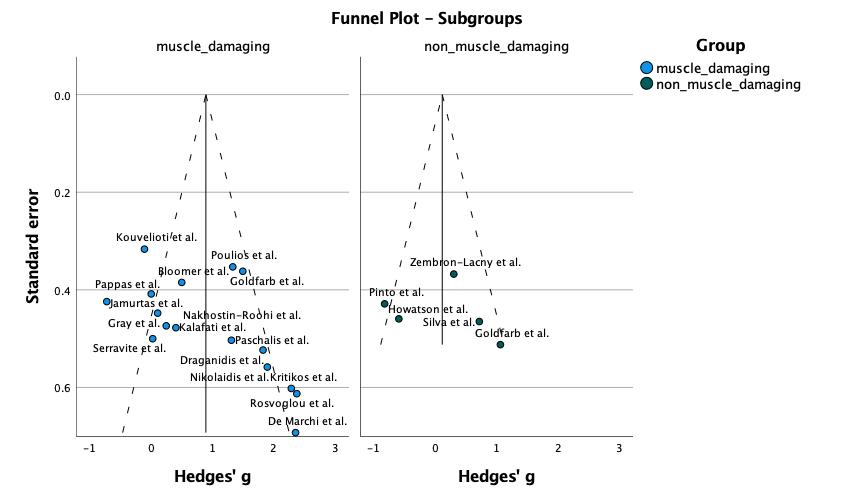 | Overall (k=24)  t=-2.325, **p=.031**  95%CI= [-4.192 – -.220]  Non-muscle-damaging (k=6)  Non-applicable  Muscle-damaging (k=18)  t=-1.974, p=.068  95%CI= [-4.025 – .167] |
| 48h post-exercise | | |
| 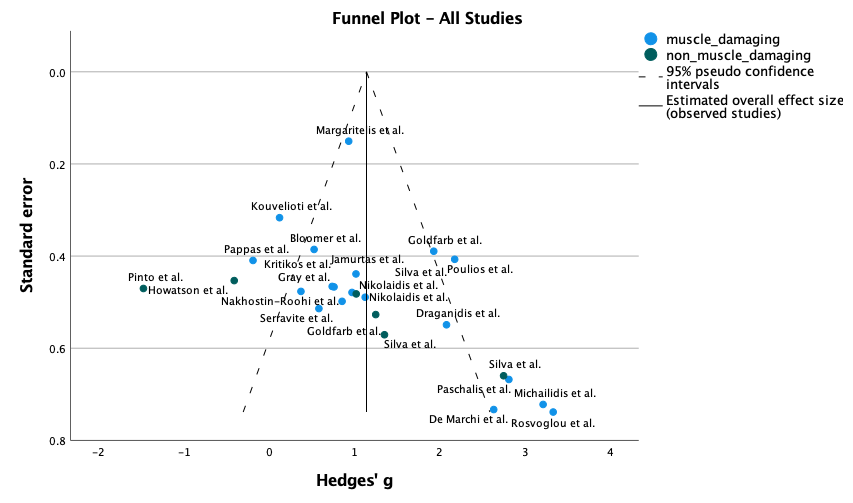 | 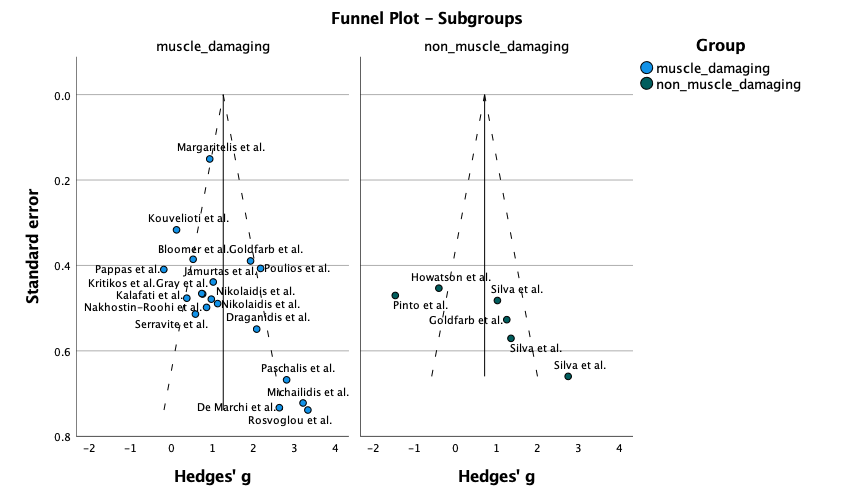 | Overall (k=27)  t=-1.666, p=.109  95%CI= [-2.703 – .291]  Non-muscle-damaging (k=6)  Non-applicable  Muscle-damaging (k=21)  t=-1.219, p=.240  95%CI= [-2.054 – .550] |
| 72h post-exercise | | |
| Fewer than 10 studies were available for this time point in total (k=9) | Fewer than 10 studies were available for each exercise type (k=9 for muscle-damaging exercise; k=0 for non-muscle-damaging) | Overall (k=9)  Non-applicable  Non-muscle-damaging (k=0)  Non-applicable  Muscle-damaging (k=9)  Non-applicable |

# Figure S1. PRISMA 2020 flow diagram for glutathione

**Identification of studies via databases and registers**

Records removed before screening:

Duplicate records removed

(n = 502)

Records marked as ineligible by automation tools (n = 0)

Records removed by title screening (n = 977)

Records identified from:

PubMed (n = 2269)

EMBASE (n = 739)

Registers (n = 0)

Overall retrieved (n = 3008)

**Identification**

Records screened

(n = 1529)

Records excluded for reliability issues

(n = 772)

Reports sought for retrieval

(n = 757)

Reports not retrieved

(n = 0)

**Screening**

Reports assessed for eligibility

(n = 757)

Reports excluded:

Reviews, Editorials, Case reports, Consensus papers, Conferences, Not fulfill the eligibility criteria (n = 716)

Studies included in review

(n = 41)

**Included**

# Figure S2. PRISMA 2020 flow diagram for F_2_-isoprostanes

**Identification of studies via databases and registers**

Records removed before screening:

Duplicate records removed

(n = 498)

Records marked as ineligible by automation tools (n = 0)

Records removed by title screening (n = 961)

Records identified from:

PubMed (n = 2389)

EMBASE (n = 761)

Registers (n = 0)

Overall retrieved (n = 3150)

**Identification**

Records screened

(n = 1691)

Records excluded for reliability issues

(n = 886)

Reports sought for retrieval

(n = 805)

Reports not retrieved

(n = 0)

**Screening**

Reports assessed for eligibility

(n = 805)

Reports excluded:

Reviews, Editorials, Case reports, Consensus papers, Conferences, Not fulfill the eligibility criteria (n = 765)

Studies included in review

(n = 40)

**Included**

# Figure S3. PRISMA 2020 flow diagram for protein carbonyls

**Identification of studies via databases and registers**

Records removed before screening:

Duplicate records removed

(n = 789)

Records marked as ineligible by automation tools (n = 0)

Records removed by title screening (n = 1330)

Records identified from:

PubMed (n = 3283)

EMBASE (n = 1021)

Registers (n = 0)

Overall retrieved (n = 4304)

**Identification**

Records screened

(n = 2185)

Records excluded for reliability issues

(n = 1054)

Reports sought for retrieval

(n = 1131)

Reports not retrieved

(n = 0)

**Screening**

Reports assessed for eligibility

(n = 1131)

Reports excluded:

Reviews, Editorials, Case reports, Consensus papers, Conferences, Not fulfill the eligibility criteria (n = 1079)

Studies included in review

(n = 52)

**Included**

# Figure S4. Risk of bias assessment with ROB2 traffic light plot

| 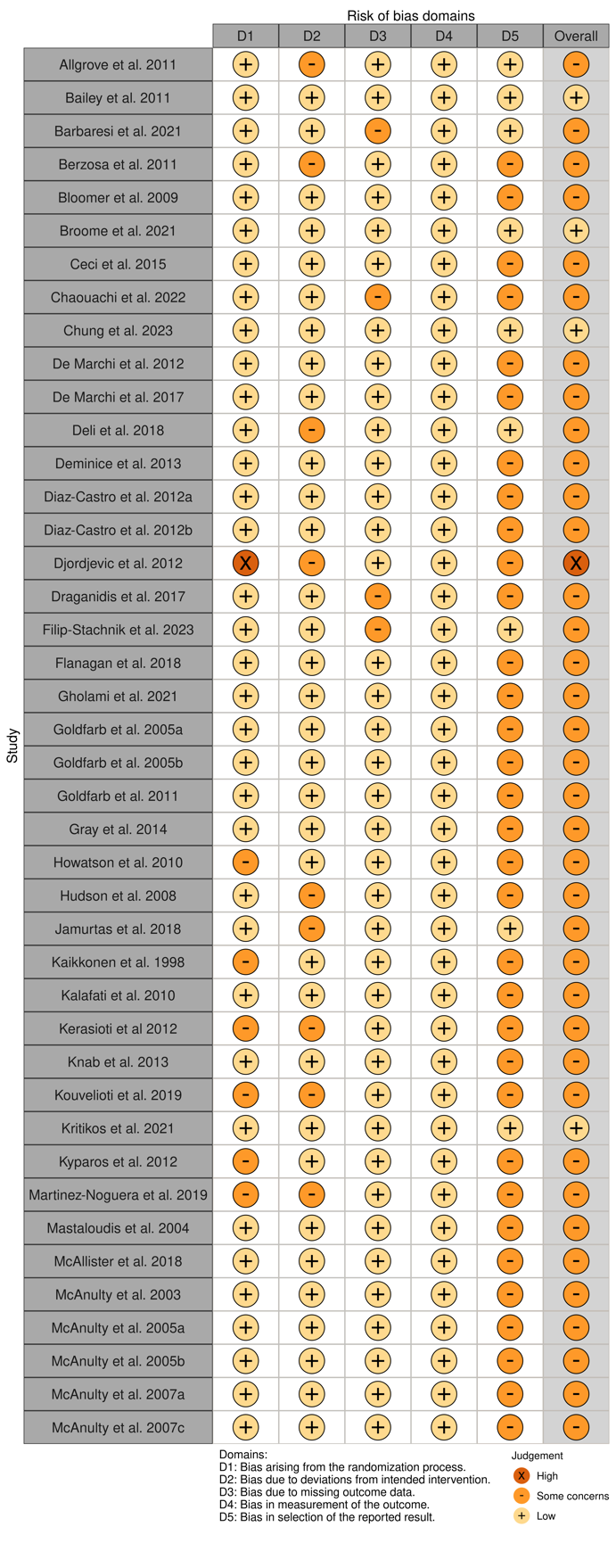 | 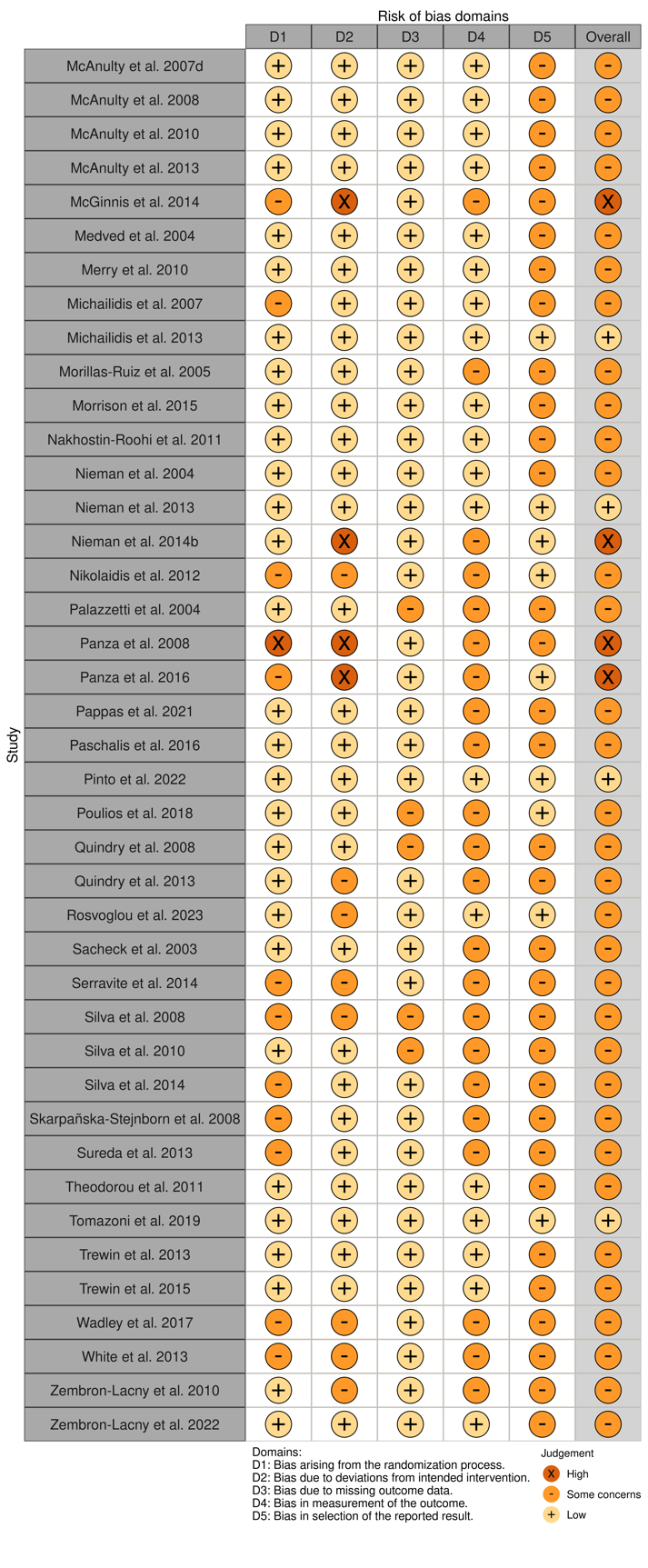 |
| --- | --- |

# Figure S5. Risk of bias assessment with ROBINS-I traffic light plot

| 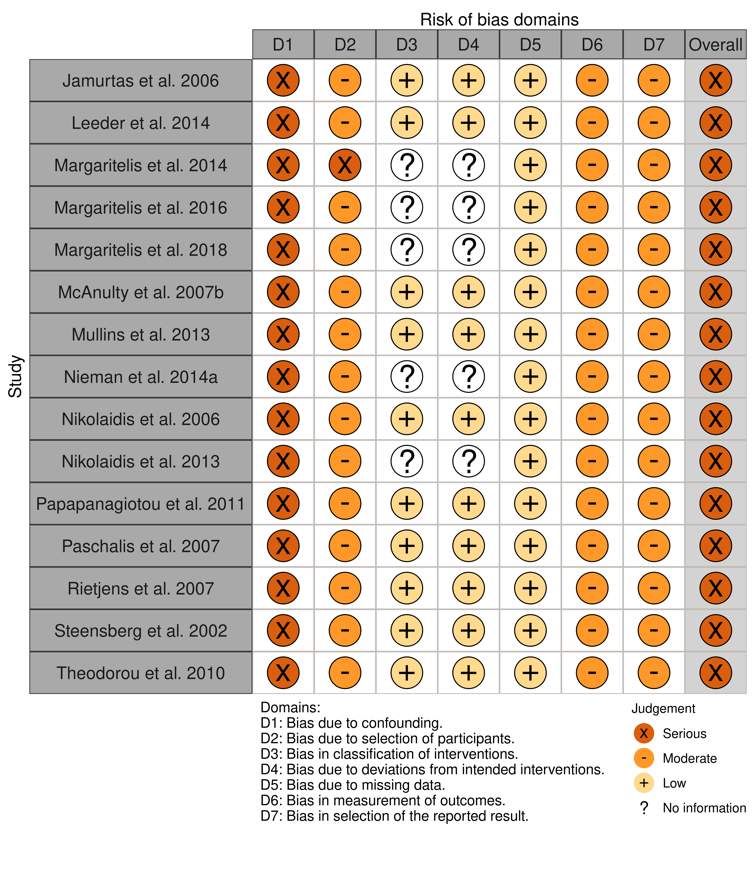 |
| --- |

# Figure S6. Subgroup analysis based on the biological specimen (i.e., erythrocyte vs. skeletal muscle) for GSH immediately post-exercise

**
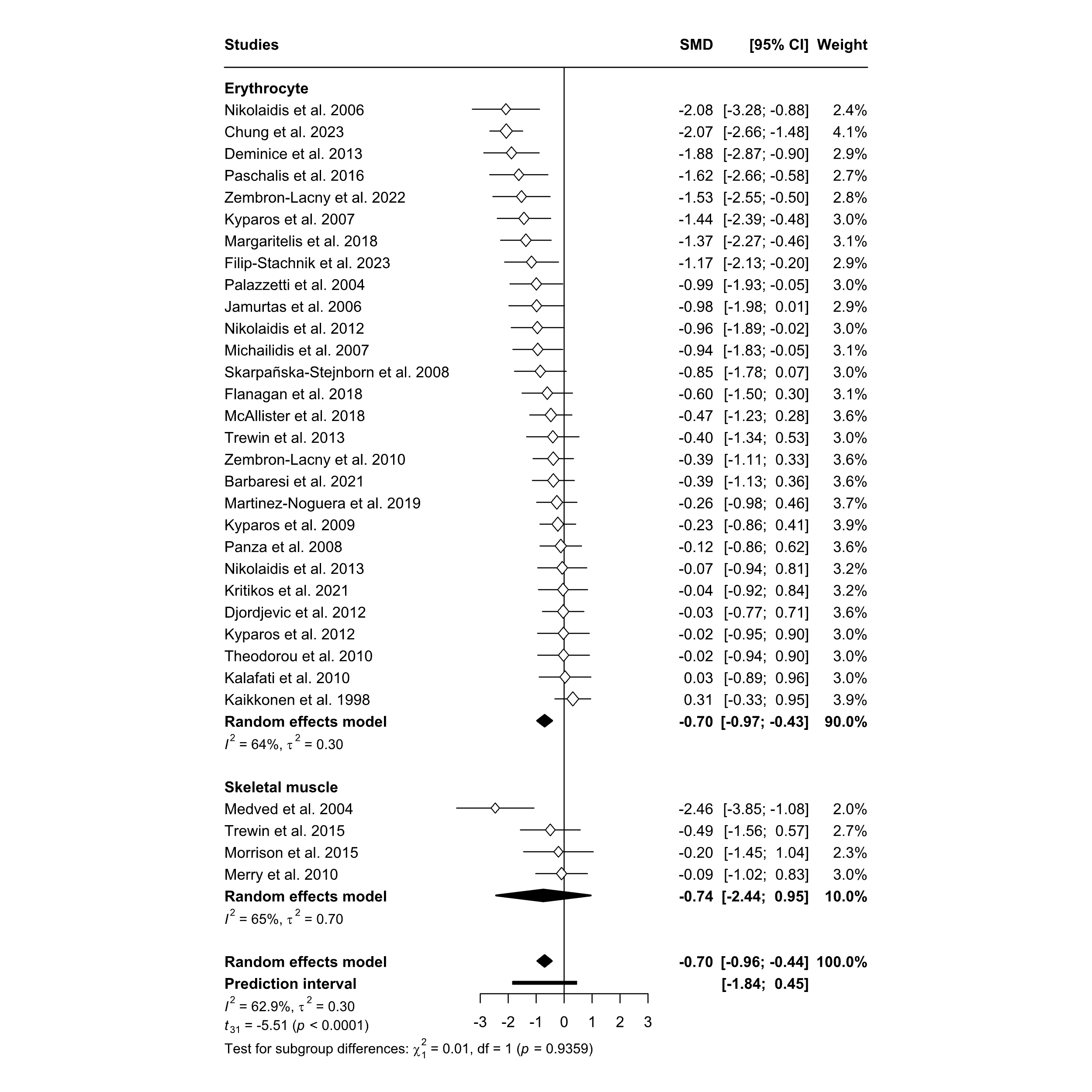
**

# Figure S7. Subgroup analysis based on the biological specimen (i.e., plasma vs. urine) for F_2_-isoprostanes immediately post-exercise


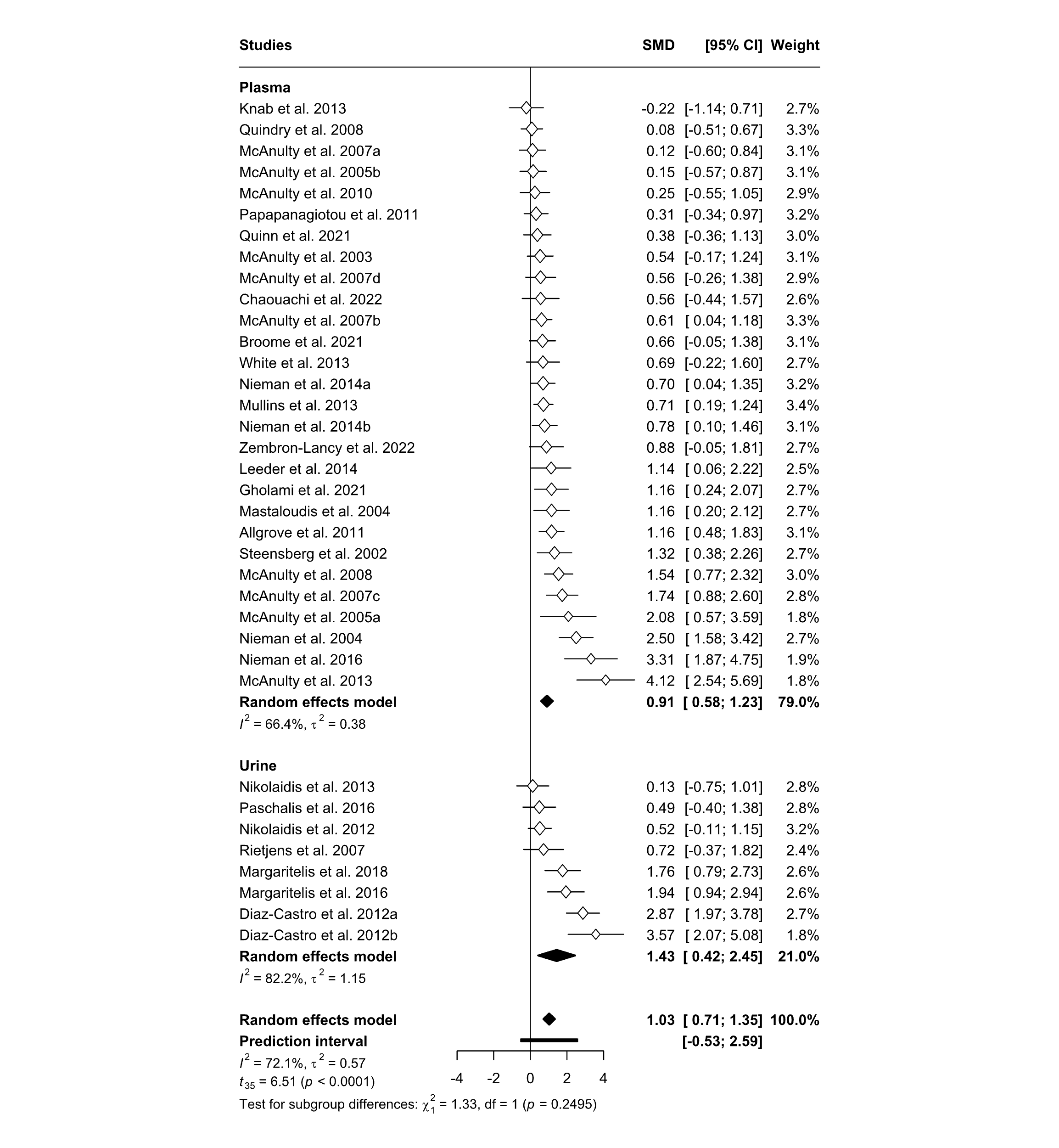

Supplement: Supplementary file 1 — Tables S1–S4, Figures S1–S7. [file EPH-9999-0-s003.docx]
